# Nectin-4 *cis*-interacts with ErbB2 and its trastuzumab-resistant splice variants, enhancing their activation and DNA synthesis

**DOI:** 10.1038/s41598-019-55460-9

**Published:** 2019-12-12

**Authors:** Shin Kedashiro, Ayumu Sugiura, Kiyohito Mizutani, Yoshimi Takai

**Affiliations:** From the Division of Pathogenetic Signaling, Department of Biochemistry and Molecular Biology, Kobe University Graduate School of Medicine, 1-5-6 Minatojima-minamimachi, Chuo-ku, Kobe, Hyogo 650-0047 Japan

**Keywords:** Growth factor signalling, Biochemistry

## Abstract

Nectin-4 cell adhesion molecule and ErbB2 tyrosine kinase receptor are upregulated in many cancers, including breast cancer, and promote cancer cell proliferation and metastasis. Using human breast cancer cell lines T47D and SUM190-PT, in which both nectin-4 and ErbB2 were upregulated, we showed here that nectin-4 *cis*-interacted with ErB2 and enhanced its dimerization and activation, followed by the activation of the phosphoinositide 3-kinase-AKT signalling pathway for DNA synthesis. The third immunoglobulin-like domain of nectin-4 *cis*-interacted with domain IV of ErbB2. This region differs from the trastuzumab-interacting region but is included in the trastuzumab-resistant splice variants of ErbB2, p95-ErbB2 and ErbB2ΔEx16. Nectin-4 also *cis*-interacted with these trastuzumab-resistant splice variants and enhanced the activation of the phosphoinositide 3-kinase-AKT signalling pathway for DNA synthesis. In addition, nectin-4 enhanced the activation of the p95-ErbB2-induced JAK-STAT3 signalling pathway, but not the ErbB2- or ErbB2ΔEx16-induced JAK-STAT3 signalling pathway. These results indicate that nectin-4 *cis*-interacts with ErbB2 and its trastuzumab-resistant splice variants and enhances the activation of these receptors and downstream signalling pathways in a novel mechanism.

## Introduction

ErbB2 is a receptor tyrosine kinase belonging to the epidermal growth factor (EGF) receptor family with four members, ErbB1, -2, -3, -4, which are also known as EGF receptor for ErbB1 and HER1, -2, -3, -4, respectively^1^. ErbB2 is frequently upregulated by its gene amplification in many types of cancers, including breast cancer, and plays a role as an oncogenic protein, which induces tumourigenesis, invasion, and metastasis^1–5^. The incidence of ErbB2-upregulated breast cancers is approximately 20–30% of total breast cancer cases^6,7^. ErbB2-targeting monoclonal antibody (mAb) drugs, such as trastuzumab (also known as Herceptin) and pertuzumab (also known as Perjeta), have been developed and used as therapeutic drugs for ErbB2-upregulated breast cancers^8^. However, two splice variants of ErbB2, p95-ErbB2 and ErbB2ΔEx16, have been identified as oncogenic proteins in trastuzumab-resistant breast cancers^9–20^, although trastuzumab-resistance to ErbB2ΔEx16 is controversial^21,22^. The incidence of p95-ErbB2- or ErbB2ΔEx16-upregulated breast cancers is 30% and 90%, respectively, of the total ErbB2-upregulated breast cancers^14,23,24^. Thus, the development of therapeutic drugs for trastuzumab-resistant breast cancers expressing such variants is awaited.

**Figure 1:**
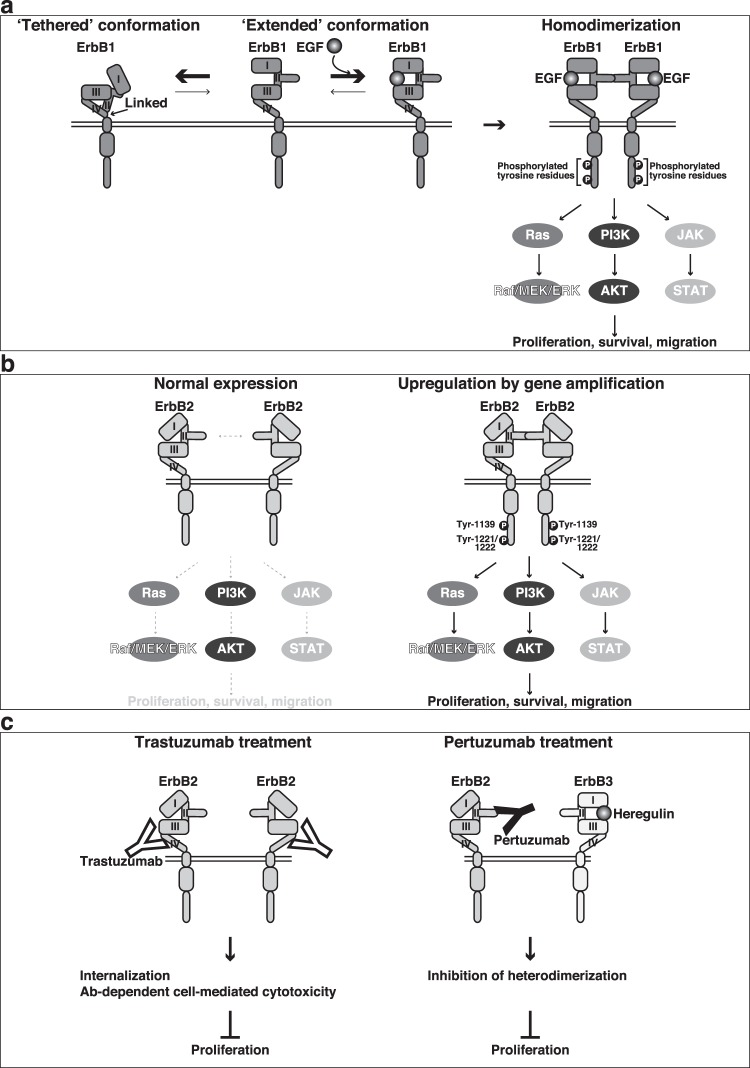
Schematic presentation of the mechanism for the homodimerization and activation of ErbB1 and ErbB2 and the inhibitory mechanism for the activation of ErbB2 by its antibodies. (**a)** A mechanism for ErbB1. (**b)** A mechanism for ErbB2. (**c)** Inhibitory mechanism for the activation of ErbB2 by trastuzumab and pertuzumab.

Of the ErbB family members, the activation mechanism has most extensively been investigated for ErbB1^25^. One EGF molecule binds to one ErbB1 molecule, resulting in its homodimerization. Crystal structure and NMR analyses have revealed that the ErbB1 molecule exists in a ‘tethered’ or an ‘extended’ structure in the absence of EGF^25,26^. In the tethered structure, the extracellular domains II and IV are linked, but in the extended structure of ErbB1, domain II, which is a binding site for another ErbB1 molecule, is released^25^ **(**Fig. 1a**)**. These two structures are in dynamic equilibrium, and the binding of one EGF molecule to domain I and domain III promotes the conversion of the tethered structure to the extended structure **(**Fig. 1a**)**. This conversion leads to the homodimerization of ErbB1, promoting the tyrosine kinase activity of each receptor, followed by the intermolecular tyrosine phosphorylation **(**Fig. 1a**)**. The Src homology 2 domain-containing protein Grb2 binds to the tyrosine-phosphorylated ErbB1 molecule and activates at least three signalling pathways, Ras-Raf-MEK-ERK, phosphoinositide 3-kinase (PI3K)-AKT, and JAK-STAT signalling pathways, for cell proliferation, survival, and migration, and their continuous abnormal activation eventually leads to cancer cell tumourigenesis, invasiveness, and metastasis^1–5,27–32^. The truncated mutant of ErbB1 is an oncogenic protein that possesses the homodimerization property even in the absence of EGF and continuously transduces cell proliferation signals^33,34^.

ErbB2 has a kinase domain in the cytoplasmic tail, but any ligand for ErbB2 has not been identified, unlike ErbB1, ErbB3, and ErbB4, which have specific ligands^1–5^. Crystallographic studies have revealed that ErbB2 exists in a distinct structure in which it exhibits an extended structure in the absence of ligands so that the loop in domain II of one ErbB2 molecule is ready to bind to that of another ErbB2 moolecule^35^ **(**Fig. 1b**)**. However, the affinity of the loop in domain II of ErbB2 for its homodimerization is low and the homodimerization of ErbB2 requires its upregulation, which is frequently induced by amplification of *ERBB2* in various cancers, including breast cancer^31^. The upregulated ErbB2-induced homodimerization enhances its tyrosine-phosphorylation and the activation of at least the same three signalling pathways as those downstream of ErbB1 for cell proliferation, survival, and migration, eventually leading to cancer cell tumourigenesis, invasiveness, and metastasis^1–5,27–32^ **(**Fig. 1b**)**. Thus, upregulated ErbB2 also serves as an oncogenic protein in this mechanism. Trastuzumab interacts with domain IV of ErbB2 and enhances its internalization, causing inhibition of the ErbB2 signalling pathway for cell proliferation, although its mode of action differs depending on cancer cell type^18^ **(**Fig. 1c**)**. Trastuzumab further targets tumour cells by antibody (Ab)-dependent cell-mediated cytotoxicity in a patient’s immune system. Pertuzumab interacts with domain II of ErbB2 and inhibits its heterodimerization with ErbB3 and activation, causing inhibition of the ErbB2 signalling pathway for cell proliferation^36,37^ **(**Fig. 1c**)**.

Nectin-4 is a cell adhesion molecule (CAM), which was originally identified by Lopez’s group^38^. It belongs to the nectin-like molecule (Necl) family with five members (Necl-1, -2, -3, -4 and -5), which comprises a superfamily with the nectin family with four members (nectin-1, -2, -3, and -4)^39–41^. These members *trans*-interact with each other in a homophilic or heterophilic manner and regulate diverse events mediated by cell-cell adhesion in various cells^39–41^. In addition, they *cis*-interact with cell surface membrane receptors, such as the platelet-derived growth factor receptor, the fibroblast growth factor receptor, the vascular endothelial growth factor receptor, the prolactin receptor, ErbB3, and ErbB4, and integrins, such as integrin αvβ3 and integrin α6β4, and regulate not only cell–cell adhesion but also cell migration, proliferation, differentiation, and survival^42–45^.

Nectin-4 is upregulated in various cancers, such as breast^46^, lung^47^, ovarian^48^, pancreatic^49^, gallbladder^50^, and gastric cancer^51^, and promotes cancer cell proliferation and metastasis^47,50,52,53^. The PI3K-AKT signalling pathway is implicated in these roles of nectin-4 through activation of the Wnt-β-catenin and Rac small G protein signalling pathways^51,54^. In addition, breast cancer cells cultured in soft agar express nectin-4 and nectin-1 that *trans*-interact each other in a heterophilic manner, and nectin-4 also *trans*-interacts in a homophilic manner^52^. These interactions enable the cancer cells to survive in an anchorage-independent manner in soft agar^52^. Nectin-4 further enables epithelial and cancer cells to resist ferroptosis by clustering each cell for survival under a matrix-detached condition^55^. The concept derived from these results for anti-cancer drugs and a diagnostic marker development by interception of nectin-4 function has now been applied^46,56–58^.

We found here that nectin-4 *cis*-interacted with ErbB2 and enhanced its homodimerization and activation in a novel mechanism, followed by the activation of the PI3K-AKT signalling pathway for DNA synthesis. We further found that nectin-4 also *cis*-interacted with the trastuzumab-resistant splice variant of ErbB2, p95-ErbB2, and a more aggressive splice variant for tumourigenesis, ErbB2ΔEx16, and enhanced the activation of the PI3K-AKT signalling pathway for DNA synthesis. In addition, nectin-4 enhanced the activation of the p95-ErbB2-induced JAK-STAT3 signalling pathway, but not the ErbB2- or ErbB2ΔEx16-induced JAK-STAT3 signalling pathway.

## Results

### *Cis*-interaction of nectin-4 with ErbB2

**Figure 2:**
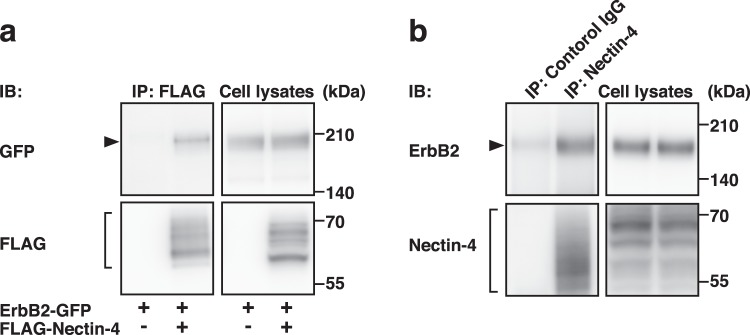
*Cis*-interaction of nectin-4 with ErbB2. (**a)** *Cis*-interaction of nectin-4 with ErbB2. HEK293E cells were co-transfected with various combinations of the indicated plasmids and cultured. The cells were detached using Accutase and cultured for 1 h in suspension. The cells were collected and lysed, and FLAG-tagged nectin-4 (FLAG-Nectin-4) was immunoprecipitated using an anti-FLAG mAb. The samples were subjected to Western blotting using the indicated Abs. (**b)** *Cis*-interaction of endogenous nectin-4 with endogenous ErbB2. SUM190-PT cells were detached using Accutase and cultured for 1 h in suspension. The cells were collected and lysed, and endogenous nectin-4 was immunoprecipitated using an anti-nectin-4 pAb. The samples were subjected to Western blotting using the indicated Abs. Arrowheads and square brackets indicate each of the proteins. The displayed blots were cropped, and the full-length blots are shown in Supplementary Fig. 4. IB, immunoblotting; IP, immunoprecipitation. Representative results from three independent experiments are shown.

We first examined whether nectin-4 *cis*-interacts with ErbB2. FLAG-tagged nectin-4 (FLAG-Nectin-4) was co-expressed with GFP-tagged ErbB2 (ErbB2-GFP) in human embryonic kidney (HEK) 293E cells. The cells were cultured in suspension to enable the detection of a possible *cis*-interaction between nectin-4 and ErbB2 on the same  plasma membrane. When FLAG-Nectin-4 was immunoprecipitated using an anti-FLAG mAb, ErbB2-GFP was co-immunoprecipitated with FLAG-Nectin-4 **(**Fig. 2a**)**. ErbB1-GFP, ErbB3-GFP, or ErbB4-GFP was not co-immunoprecipitated with FLAG-Nectin-4 under the same assay condition **(**Supplementary Fig. 1b–e**)**. When endogenous nectin-4 was immunoprecipitated using an anti-nectin-4 polyclonal Ab (pAb) in SUM190-PT breast cancer cells, endogenous ErbB2 was co-immunoprecipitated with endogenous nectin-4 **(**Fig. 2b**)**. In this cell line, nectin-4 and nectin-1 were expressed, but nectin-2, nectin-3, Necl-1, Necl-2, Necl-3, Necl-4, or Necl-5, was not detected **(**Supplementary Fig. 1a**)**. ErbB1, ErbB2, ErbB3, and ErbB4 were also expressed. In addition, endogenous nectin-4 and ErbB2 were co-localized at cell-cell adhesion sites in T47D and SUM190-PT cells (Supplementary Fig. 1f**)**. These results indicate that nectin-4 *cis*-interacts specifically with ErbB2 among the ErbB family members and that this *cis*-interaction occurs on the same plasma membrane.

### Enhancement of the homodimerization and tyrosine-phosphorylation of ErbB2 by nectin-4

**Figure 3:**
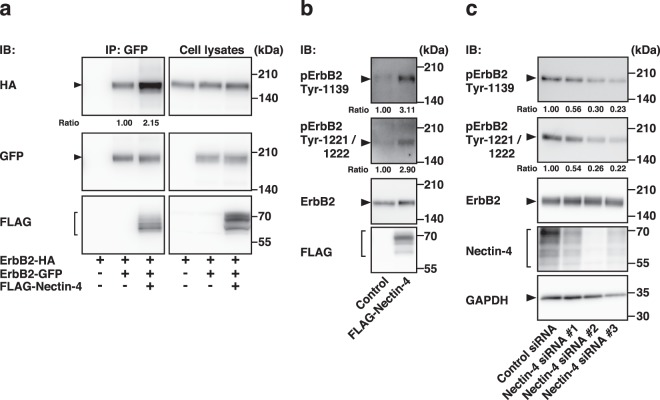
Enhancement of the homodimerization and tyrosine-phosphorylation of ErbB2 by nectin-4. (**a)** Enhancement of the homodimerization of ErbB2 by nectin-4. HEK293E cells were co-transfected with various combinations of the indicated plasmids, and the cells were detached using Accutase and cultured for 1 h in suspension. The cells were collected and lysed, and GFP-tagged ErbB2 (ErbB2-GFP) was immunoprecipitated using an anti-GFP pAb. The samples were subjected to Western blotting using the indicated Abs. Ratio represents the band intensities of the co-immunoprecipitaeted HA-tagged ErbB2 (ErbB2-HA) that were normalized to those of the immunoprecipitated ErbB2-GFP, and the normalized value of the both ErbB2-HA and ErbB2-GFP, but not FLAG-tagged nectin-4 (FLAG-Nectin-4), expressing cells was set as 1.00. (**b)** Enhancement of the tyrosine-phosphorylation of ErbB2 by nectin-4. T47D cells stably expressing FLAG-Nectin-4 were serum-starved for 24 h, and the samples were subjected to Western blotting using the indicated Abs. **c** Reduction of the tyrosine-phosphorylation of ErbB2 by knockdown of *NECTIN4*. SUM190-PT cells were transfected with a control siRNA or *NECTIN4* siRNAs. The cells were serum-starved for 24 h, and the samples were subjected to Western blotting using the indicated Abs. Ratio represents the band intensities of the phospho-ErbB2 on Tyr-1139 or Tyr-1221/1222 that were normalized to those of the total ErbB2, and the normalized value of the control cells was set as 1.00. Arrowheads and square brackets indicate each of the proteins. The displayed blots were cropped, and the full-length blots are shown in Supplementary Fig. 5. IB, immunoblotting; IP, immunoprecipitation. pErbB2, phospho-ErbB2. Representative results from three independent experiments are shown.

We then examined whether nectin-4 affects the homodimerization of ErbB2. HA-tagged ErbB2 (ErbB2-HA) and ErbB2-GFP were co-expressed with FLAG-Nectin-4 or with FLAG as control in HEK293E cells. When ErbB2-GFP was immunoprecipitated with an anti-GFP pAb, ErbB2-HA was co-immunoprecipitated with ErbB2-GFP and FLAG-Nectin-4, but not FLAG, and the amount of ErbB2-HA in the cells co-expressing FLAG-Nectin-4 was more than that co-immunoprecipitated with ErbB2-GFP in the cells co-expressing FLAG **(**Fig. 3a**)**. These results indicate that nectin-4 *cis*-interacts with ErbB2 and that this *cis*-interaction enhances its homodimerization.

The homodimerization of ErbB2 induces the tyrosine-phosphorylation of ErbB2 intermolecularly at several tyrosine residues including 1139, 1221, and 1222^59–61^. Using mAbs, one of which recognizes phosphorylated tyrosine residue at 1139 and  the other of which  recognizes both phosphorylated tyrosine residues at 1221 and 1222, we examined whether the nectin-4-enhanced homodimerization of ErbB2 enhances the phosphorylation of these tyrosine residues. For this purpose, we used T47D breast cancer cells, which expressed both nectin-4 and ErbB2 at much lower levels than SUM190-PT cells **(**Supplementary Fig. 1a**)**. In this cell line, nectin-1 and Necl-2, but not nectin-2, nectin-3, Necl-1, Necl-3, Necl-4, or Necl-5, were detected. The phosphorylation of tyrosine residues at 1139, 1221, and 1222 was enhanced in the T47D cells stably expressing FLAG-Nectin-4 compared with that in the control cells **(**Fig. 3b**)**. Conversely, the phosphorylation of these tyrosine residues was reduced by the siRNA-induced knockdown of *NECTIN4* in SUM190-PT cells **(**Fig. 3c**)**. The reduction of nectin-4 by the siRNA-induced knockdown was confirmed by Western blotting **(**Fig. 3c**)**. These results indicate that nectin-4 enhances the homodimerization of ErbB2, which leads to the phosphorylation of its tyrosine residues at 1139, 1221, and 1222.

### Selective enhancement of the activation of the PI3K-AKT signalling pathway by nectin-4

**Figure 4:**
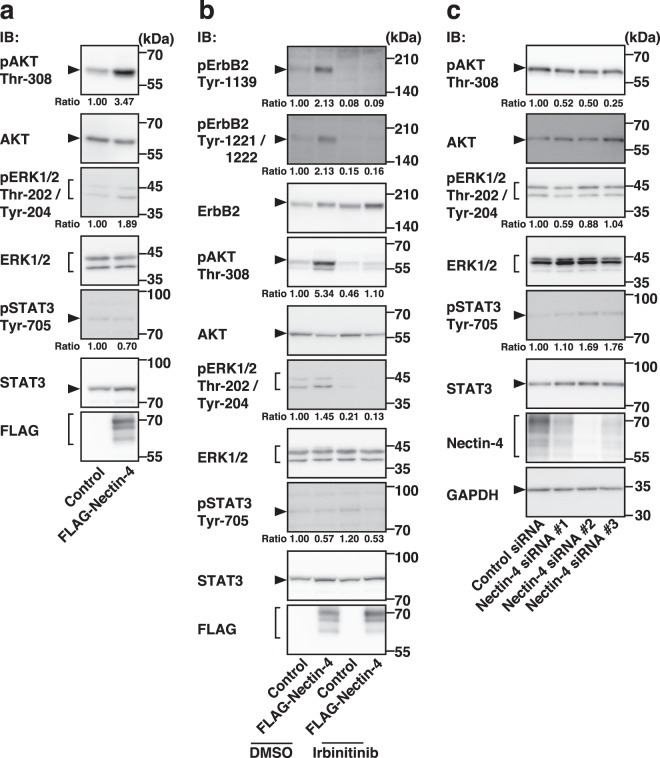
Selective enhancement of the activation of the PI3K-AKT signalling pathway by nectin-4. (**a)** Selective enhancement of the activation of the PI3K-AKT signalling pathway by nectin-4. T47D cells stably expressing FLAG-tagged nectin-4 (FLAG-Nectin-4) were serum-starved for 24 h, and the samples were subjected to Western blotting using the indicated Abs. **(b)** Reduction of the threonine-phosphorylation of AKT and threonine- and tyrosine-phosphorylation of ERK1/2 by an ErbB2 inhibitor. T47D cells stably expressing FLAG-Nectin-4 were serum-starved and treated with the ErbB2 inhibitor irbinitinib at 1 μM. The assay was carried out as in (**a**). (**c)** Reduction of the threonine-phosphorylation of AKT by the knockdown of *NECTIN4*. SUM190-PT cells were transfected with a control siRNA or *NECTIN4* siRNAs. The assay was carried out as (**a**). Ratio represents the band intensities of each phosphorylated protein on the indicated tyrosine and threonine residues that were normalized to those of each total protein, and the normalized value of the control cells (DMSO-, but not Irbinitinib-, treated cells **(b)** or control siRNA cells **(c)**) was set as 1.00. Arrowheads and square brackets indicate each of the proteins. The displayed blots were cropped, and the full-length blots are shown in Supplementary Figs. 6 and 7. IB, immunoblotting; pAKT, phospho-AKT; pErbB2, phospho-ErbB2; pERK1/2, phospho-ERK1/2; pSTAT3, phospho-STAT3. Representative results from three independent experiments are shown.

The tyrosine-phosphorylation of ErbB2 leads to the activation of the PI3K-AKT, Ras-Raf-MEK-ERK, and JAK-STAT signalling pathways^1–5,27–30,32^. We therefore examined the effects of nectin-4 on the activation of these signalling pathways. The threonine-phosphorylation of AKT was markedly enhanced in the T47D cells stably expressing FLAG-Nectin-4 compared with that in the control cells, whereas the threonine- and tyrosine-phosphorylation of ERK1/2 or the tyrosine-phosphorylation of STAT3 was not significantly enhanced in the T47D cells stably expressing FLAG-Nectin-4 compared with that in the control cells **(**Fig. 4a**)**. The threonine-phosphorylation of AKT was inhibited by the tyrosine kinase inhibitor for ErbB2, irbinitinib, in the T47D cells stably expressing FLAG-Nectin-4 and in the control cells **(**Fig. 4b**)**. Conversely, the threonine-phosphorylation of AKT was reduced in the SUM190-PT cells in which endogenous nectin-4 was knocked down compared with that in the control cells **(**Fig. 4c**)**. These results indicate that nectin-4 mainly enhances the ErbB2-mediated PI3K-AKT signalling pathway, but not the Ras-Raf-MEK-ERK1/2 signalling pathway or the JAK-STAT3 signalling pathway.

### Enhancement of DNA synthesis by nectin-4 through the ErbB2-mediated PI3K-AKT signalling pathway

**Figure 5:**
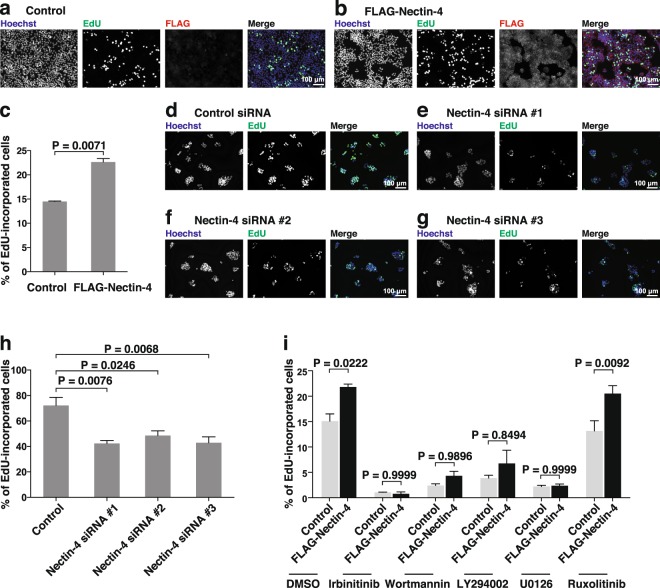
Enhancement of DNA synthesis by nectin-4 through the ErbB2-mediated PI3K-AKT signalling pathway. (**a–c)** Enhancement of DNA synthesis by nectin-4. T47D control cells (**a**) or T47D cells stably expressing FLAG-tagged nectin-4 (FLAG-Nectin-4) (**b**) were serum-starved and treated with medium containing 10 μM EdU for 12 h. After washout of the medium, the cells were cultured in the absence of serum and stained with an anti-DDDDK mAb, Hoechst33342, and EdU staining reagents. The number of EdU-incorporated cells was counted by microscopy and Hybrid Cell Counter software (**c**). (**d–h)** Reduction of DNA synthesis by the knockdown of *NECTIN4*. SUM190-PT cells were transfected with a control siRNA (**d**) or *NECTIN4* siRNAs (**e–g**), and the number of EdU-incorporated cells was counted by microscopy and Hybrid Cell Counter software (**h**). The assay was carried out as in (**a–c**). (**i)** Reduction of DNA synthesis by ErbB2, PI3K, and MEK inhibitors, but not a JAK inhibitor. T47D cells stably expressing FLAG-Nectin-4 were serum-starved and treated with the ErbB2 inhibitor irbinitinib at 1 μM, the PI3K inhibitor wortmannin at 1 μM or the PI3K inhibitor LY294002 at 50 μM, the MEK inhibitor U0126 at 10 μM, or the JAK1 and JAK2 inhibitor ruxolitinib at 1 μM. The assay was carried out as in (**a–c**). Bars indicate the means ± S.E. of three independent experiments. The actual P values for each test are shown in each figure. Scale bars, 100 μm. Representative results (images) from three independent experiments are  shown.

The enhancement of the phosphorylation of tyrosine residues of ErbB2 induces cell proliferation through the PI3K-AKT, Ras-Raf-MEK-ERK, and JAK-STAT signalling pathways^1–5,27–30,32^. We estimated cell proliferation by measuring the incorporation of the DNA base analogue 5-ethynyl-2′-deoxyuridine (EdU) into DNA. We first examined whether nectin-4 enhances the ErbB2-mediated DNA synthesis. The incorporation of EdU into the DNA of the T47D cells stably expressing FLAG-Nectin-4 was enhanced compared with that in the control cells **(**Fig. 5a–c**)**. The incorporation of EdU into the DNA of the T47D cells stably expressing FLAG-Nectin-4 and the control cells was inhibited by irbinitinib **(**Fig. 5i**)**. In addition, the incorporation of EdU into the DNA of the SUM190-PT cells in which endogenous nectin-4 was knocked down was reduced compared with that in the control cells **(**Fig. 5d–h**)**. These results indicate that nectin-4 enhances the ErbB2-mediated DNA synthesis.

We then examined which signalling pathway, PI3K-AKT, Ras-Raf-MEK-ERK1/2, and/or JAK-STAT3 signalling pathways, is involved in this nectin-4-enhanced ErbB2-mediated DNA synthesis. The incorporation of EdU into DNA in the T47D cells stably expressing FLAG-Nectin-4 and the control cells was inhibited by the PI3K inhibitors wortmannin and LY294002 and the MEK inhibitor U0126, but not by the JAK1/2 inhibitor ruxolitinib **(**Fig. 5i**)**. These results indicate that nectin-4 enhances the ErbB2-mediated DNA synthesis through the activation of the PI3K-AKT signalling pathway, and that the nectin-4-independent ErbB2-mediated activation of the Ras-Raf-MEK-ERK1/2 pathway, but not the JAK-STAT3 pathway, is also involved in the ErbB2-mediated DNA synthesis.

### The *cis*-interaction of the third immunoglobulin-like domain of nectin-4 with domain IV of ErbB2

We next attempted to determine the ErbB2-interacting domain of nectin-4. For this purpose, FLAG-tagged mutants of nectin-4 in which the cytoplasmic domain was deleted (FLAG-Nectin-4-immunoglobulin (Ig)1/2/3-transmembrane (TM)), the cytoplasmic domain and first Ig-like domains were deleted (FLAG-Nectin-4-Ig2/3-TM), the cytoplasmic domain and the first and second Ig-like domains were deleted (FLAG-Nectin-4-Ig3-TM), and the first, second, and third Ig-like domains were deleted (FLAG-Nectin-4-TM-cytoplasmic (CP)), were prepared **(**Supplementary Fig. 2a**)**, and full-length FLAG-tagged or each of these nectin-4 mutants was co-expressed with ErbB2-GFP in HEK293E cells. When each of the FLAG-tagged nectin-4 mutants was immunoprecipitated using the anti-FLAG mAb, ErbB2-GFP was co-immunoprecipitated with full-length nectin-4 (FLAG-Nectin-4-FL), FLAG-Nectin-4-Ig1/2/3-TM, FLAG-Nectin-4-Ig2/3-TM, and FLAG-Nectin-4-Ig3-TM, but not with FLAG-Nectin-4-TM-CP **(**Supplementary Fig. 2b**)**. These results indicate that nectin-4 *cis*-interacts with ErbB2 through the third Ig-like domain, although it cannot be excluded that other domains are also involved in the *cis*-interaction with ErbB2.

We then examined the nectin-4-interacting domain of ErbB2. For this purpose, GFP-tagged mutants of ErbB2, in which the cytoplasmic domain was deleted (ErbB2-1/2/3/4-TM-GFP), the cytoplasmic domain and domain I were deleted (ErbB2-2/3/4-TM-GFP), the cytoplasmic domain, domain I, and domain II were deleted (ErbB2-3/4-TM-GFP), and the cytoplasmic domain, domain I, domain II, and domain III were deleted (ErbB2-4-TM-GFP), were prepared **(**Supplementary Fig. 2a**)** and full-length GFP-tagged or each of these ErbB2 mutants was co-expressed with FLAG-Nectin-4 in HEK293E cells. When each of the GFP-tagged ErbB2 mutants was immunoprecipitated using the anti-GFP pAb, FLAG-Nectin-4 was co-immunoprecipitated with full-length ErbB2 (ErbB2-FL-GFP), ErbB2-1/2/3/4-TM-GFP, ErbB2-2/3/4-TM-GFP, ErbB2-3/4-TM-GFP, and ErbB2-4-TM-GFP **(**Supplementary Fig. 2c**)**. Essentially the same results were obtained in the reciprocal immunoprecipitation assay **(**Supplementary Fig. 2d–h**)**. These results indicate that ErbB2 *cis*-interacts with nectin-4 through domain IV, although it cannot be excluded that other domains are also involved in the *cis*-interaction with nectin-4.

### Inhibition of the *cis*-interaction of nectin-4 with ErbB2 by the recombinant protein of the third Ig-like domain of nectin-4

We then examined whether a recombinant third Ig-like domain of nectin-4 shows an inhibitory effect on the *cis*-interaction of full-length nectin-4 with ErbB2 as schematically shown in Supplementary Fig. 3a–c, because this domain was a minimal interacting region with ErbB2. Recombinant FLAG-tagged extracellular region of nectin-4 (rec-FLAG-Nectin-4-Ig1/2/3) or recombinant FLAG-tagged third Ig-like domain of nectin-4 (rec-FLAG-Nectin-4-Ig3) was expressed in HEK293E cells and affinity-purified **(**Supplementary Fig. 3d**)**. ErbB2-GFP was immunopurified from ErbB2-GFP-transfected HEK293E cells using anti-GFP-pAb-conjugated beads. When the fixed amount of rec-FLAG-Nectin-4-Ig1/2/3 or rec-FLAG-Nectin-4-Ig3 was incubated with the ErbB2-GFP-immobilized beads, both rec-FLAG-Nectin-4-Ig1/2/3 and rec-FLAG-Nectin-4-Ig3 were co-immunoprecipitated with ErbB2-GFP **(**Supplementary Fig. 3a,b,e**)**. When both recombinant proteins were simultaneously incubated with ErbB2-GFP, the amount of rec-FLAG-Nectin-4-Ig1/2/3 co-precipitated with ErbB2-GFP was reduced in the presence of rec-FLAG-Nectin-4-Ig3, and *vice versa* **(**Supplementary Fig. 3c,e**)**. This result indicates that the third Ig-like domain of nectin-4 is required for the *cis*-interaction of full-length nectin-4 with ErbB2. Together with the above result showing that the third Ig-like domain of nectin-4 alone interacts with ErbB2, these results indicate that the third Ig-like domain of nectin-4 is required and sufficient for its *cis*-interaction with ErbB2.

### Enhancement of the homodimerization and tyrosine-phosphorylation of ErbB2, activation of AKT, and DNA synthesis by the third Ig-like domain of nectin-4

**Figure 6:**
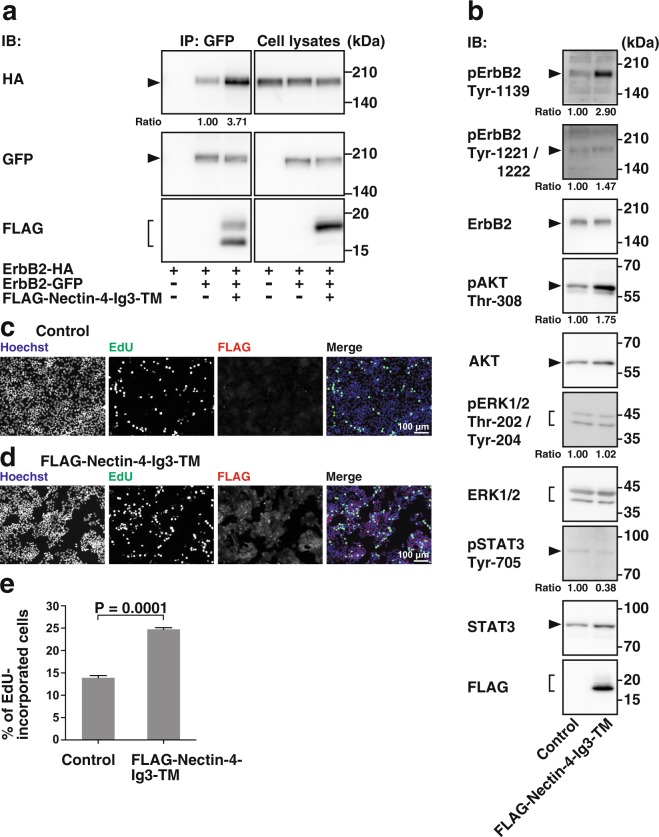
Enhancement of the homodimerization and tyrosine-phosphorylation of ErbB2, AKT activation, and DNA synthesis by the third immunoglobulin-like domain of nectin-4. (**a)** Enhancement of the homodimerization of ErbB2 by the third immunoglobulin (Ig)-like domain of nectin-4. HEK293E cells were co-transfected with various combinations of the indicated plasmids and cultured in suspension. GFP-tagged ErbB2 (ErbB2-GFP) was immunoprecipitated using the anti-GFP pAb. The samples were subjected to Western blotting using the indicated Abs. Ratio represents the band intensities of the co-immunoprecipitated HA-tagged ErbB2 (ErbB2-HA) that were normalized to those of the immunoprecipitated ErbB2-GFP, and the normalized value of the both ErbB2-HA and ErbB2-GFP, but not FLAG-tagged third Ig-like domain of nectin-4 (FLAG-Nectin-4-Ig3-TM), expressing cells was set as 1.00. (**b)** Enhancement of the AKT activation by the third Ig-like domain of nectin-4. T47D cells stably expressing FLAG-Nectin-4-Ig3-TM were serum-starved for 24 h, and the samples were subjected to Western blotting using the indicated Abs. Ratio represents the band intensities of each phosphorylated protein on the indicated tyrosine and threonine residues that were normalized to those of each total protein, and the normalized value of the control cells was set as 1.00. Arrowheads and square brackets indicate each of the proteins. The displayed blots were cropped, and the full-length blots are shown in Supplementary Fig. 8. Representative results from three independent experiments are shown. (**c–e)** T47D control cells (**c**) or T47D cells stably expressing FLAG-Nectin-4-Ig3-TM (**d**) were serum-starved and treated with medium containing 10 μM EdU for 12 h. After washout of the medium, the cells were cultured in the absence of serum and stained with the anti-DDDDK mAb, Hoechst33342, and EdU staining reagents. The number of EdU-incorporated cells was counted by microscopy and Hybrid Cell Counter software (**e**). Bars indicate the means ± S.E. of three independent experiments. The actual P values for each test are shown in each figure. Scale bars, 100 μm IB, immunoblotting; IP, immunoprecipitation. Representative results (images) from three independent experiments are  shown.

We then examined whether the third Ig-like domain of nectin-4 enhances the homodimerization of ErbB2. ErbB2-HA and ErbB2-GFP were co-expressed with FLAG-Nectin-4-Ig3-TM or FLAG as control in HEK293E cells. When ErbB2-GFP was immunoprecipitated with the anti-GFP pAb, ErbB2-HA was co-immunoprecipitated with ErbB2-GFP and FLAG-Nectin-4-Ig3-TM, but not FLAG, and the amount of ErbB2-HA from the cells co-expressing FLAG-Nectin-4-Ig3-TM was more than that co-immunoprecipitated with ErbB2-GFP from the control cells **(**Fig. 6a**)**. These results indicate that the *cis*-interaction of the third Ig-like domain of nectin-4 with ErbB2 enhances its homodimerization.

We further examined whether the third Ig-like domain of nectin-4 enhances the tyrosine-phosphorylation of ErbB2, activation of AKT, and DNA synthesis. For this purpose, we established a cell line stably expressing FLAG-Nectin-4-Ig3-TM in T47D cells. The tyrosine-phosphorylation of ErbB2 and the threonine-phosphorylation of AKT were enhanced in the T47D cells stably expressing FLAG-Nectin-4-Ig3-TM compared with those in the control cells, whereas the threonine- and tyrosine-phosphorylation of ERK1/2 or the tyrosine-phosphorylation of STAT3 was not significantly enhanced in the T47D cells stably expressing FLAG-Nectin-4-Ig3-TM compared with those in the control cells **(**Fig. 6b**)**. The incorporation of EdU into the T47D cells stably expressing FLAG-Nectin-4-Ig3-TM was enhanced compared with that in the control cells **(**Fig. 6c–e**)**. These results collectively indicate that the *cis*-interaction of the third Ig-like domain of nectin-4 with ErbB2 enhances the homodimerization, leading to the activation of ErbB2 and AKT and DNA synthesis, and that the cytoplasmic region of nectin-4 is not required for these reactions.

### *Cis*-interaction of nectin-4 with trastuzumab-resistant variants of ErbB2 and enhancement of their signalling pathways for DNA synthesis

**Figure 7:**
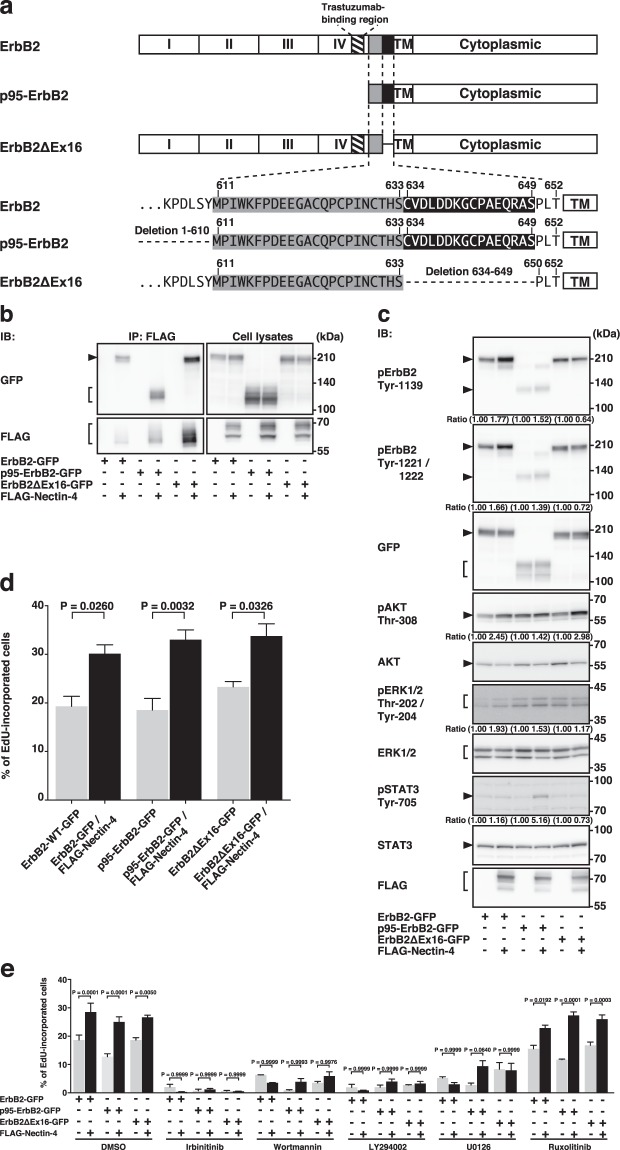
*Cis*-interaction of nectin-4 with trastuzumab-resistant variants of ErbB2 and enhancement of their signalling pathways for DNA synthesis. (**a)** Schematic diagram and amino acid sequences of ErbB2 splice variants. (**b)** *Cis*-interaction of nectin-4 with ErbB2 splice variants. HEK293E cells were co-transfected with various combinations of the indicated plasmids and cultured in suspension. FLAG-tagged nectin-4 (FLAG-Nectin-4) was immunoprecipitated using the anti-FLAG mAb. The samples were subjected to Western blotting using the indicated Abs. (**c)** Enhancement of the tyrosine-phosphorylation of ErbB2 splice variants and the PI3K-AKT signalling pathway by nectin-4. T47D cells stably expressing ErbB2 splice variants or T47D cells stably expressing GFP-tagged ErbB2 splice variants with FLAG-Nectin-4 were serum-starved for 24 h, and the samples were subjected to Western blotting using the indicated Abs. Ratio represents the band intensities of each phosphorylated protein on the indicated tyrosine and threonine residues that were normalized to those of each total protein, and the normalized value of each control cell (GFP-tagged ErbB2 and its splice variants, but not FLAG-Nectin-4, expressing cells) was set as 1.00. Arrowheads and square brackets indicate each of the proteins. The displayed blots were cropped, and the full-length blots are shown in Supplementary Fig. 9. Representative results from three independent experiments are shown. (**d)** Enhancement of DNA synthesis by nectin-4 in ErbB2 splice variant-expressing cells. T47D cells stably expressing ErbB2 splice variants or T47D cells stably expressing GFP-tagged ErbB2 splice variants with FLAG-Nectin-4 were serum-starved and treated with medium containing 10 μM EdU for 12 h. After washout of the medium, the cells were cultured in the absence of serum and stained with Hoechst33342, and EdU staining reagents. The number of EdU-incorporated cells was counted by microscopy and Hybrid Cell Counter software. (**e)** Reduction of DNA synthesis by the ErbB2, PI3K, and MEK inhibitors, but not the JAK inhibitor in ErbB2 splice variant-expressing cells. T47D cells stably expressing ErbB2 splice variants or T47D cells stably expressing GFP-tagged ErbB2 splice variants with FLAG-Nectin-4 were serum-starved and treated with the ErbB2 inhibitor irbinitinib at 1 μM, the PI3K inhibitor wortmannin at 1 μM or the PI3K inhibitor LY294002 at 50 μM, the MEK inhibitor U0126 at 10 μM, or the JAK1 and JAK2 inhibitor ruxolitinib at 1 μM. The assay was carried out as in (**d**). Bars indicate the means ± S.E. of three independent experiments. The actual P values for each test are shown in each figure. pAKT, phospho-AKT; pErbB2, phospho-ErbB2; pERK1/2, phospho-ERK1/2; pSTAT3, phospho-STAT3; IB, immunoblotting; IP, immunoprecipitation.

The ErbB2 splice variants, p95-ErbB2 and ErbB2ΔEx16, were shown to be trastuzumab-resistant in breast cancers^13,15,17^, although the trastuzumab-resistance to ErbB2ΔEx16 is controversial^21,22^. The schematic diagrams and amino acid sequences of these variants are shown in Fig. 7a. We examined whether nectin-4 *cis*-interacts with these variants. FLAG-Nectin-4 was co-expressed with p95-ErbB2-GFP or ErbB2ΔEx16-GFP in HEK293E cells. The cells were cultured in suspension. When FLAG-Nectin-4 was immunoprecipitated using the anti-FLAG mAb, both p95-ErbB2-GFP and ErbB2ΔEx16-GFP were co-immunoprecipitated with it to a similar extent **(**Fig. 7b**)**. These results indicate that nectin-4 *cis*-interacts with p95-ErbB2 and ErbB2ΔEx16 as well as ErbB2.

We next examined whether the *cis*-interaction of nectin-4 with p95-ErbB2 and ErbB2ΔEx16 enhances their tyrosine phosphorylation and AKT activation. For this purpose, we established cell lines stably co-expressing FLAG-Nectin-4 and either p95-ErbB2-GFP or ErbB2ΔEx16-GFP in T47D cells. As a control, we also established cell lines stably expressing either of these variants in T47D cells. The phosphorylation of the tyrosine residues of p95-ErbB2-GFP that are equivalent to tyrosine residues at 1139, 1221, and 1222 of wild-type ErbB2, and the threonine-phosphorylation of AKT and the tyrosine-phosphorylation of STAT3 were enhanced in the T47D cells stably expressing FLAG-Nectin-4 and p95-ErbB2-GFP compared with that in the control cells, whereas the threonine- and tyrosine-phosphorylation of ERK1/2 was not significantly enhanced in the T47D cells stably expressing FLAG-Nectin-4 and p95-ErbB2-GFP compared with that in the control cells **(**Fig. 7c**)**. The threonine-phosphorylation of AKT, but not the phosphorylation of tyrosine residues of ErbB2ΔEx16-GFP that are equivalent to tyrosine residues at 1139, 1221, and 1222 of wild-type ErbB2, the threonine- and tyrosine-phosphorylation of ERK1/2, or the tyrosine-phosphorylation of STAT3, was enhanced in the T47D cells stably expressing FLAG-Nectin-4 and ErbB2ΔEx16-GFP compared with those in the control cells **(**Fig. 7c**)**. The incorporation of EdU into the T47D cells stably co-expressing FLAG-Nectin-4 and either p95-ErbB2-GFP or ErbB2ΔEx16-GFP was enhanced compared with that in the control cells **(**Fig. 7d**)**. The incorporation of EdU into DNA in the T47D cells stably expressing FLAG-Nectin-4 and p95-ErbB2-GFP or ErbB2ΔEx16-GFP and the control cells expressing p95-ErbB2-GFP or ErbB2ΔEx16-GFP alone was inhibited by wortmannin, LY294002, and U0126, but not by ruxolitinib **(**Fig. 7e**)**. These results collectively indicate that nectin-4 *cis*-interacts with not only ErbB2 but also p95-ErbB2 and ErbB2ΔEx16, that nectin-4 enhances the p95-ErbB2- and ErbB2ΔEx16-mediated DNA synthesis through the activation of the PI3K-AKT signalling pathway, and that the nectin-4-independent p95-ErbB- and ErbBΔEx16-mediated activation of the Ras-Raf-MEK-ERK1/2 signalling pathway, but not the JAK-STAT3 signalling pathway, is also involved in the p95-ErbB- and ErbBΔEx16-mediated DNA synthesis.

### Schematic diagrams for the modes of action of nectin-4 on the signalling pathways of ErbB2 and its trastuzumab-resistant variants for DNA synthesis

**Figure 8:**
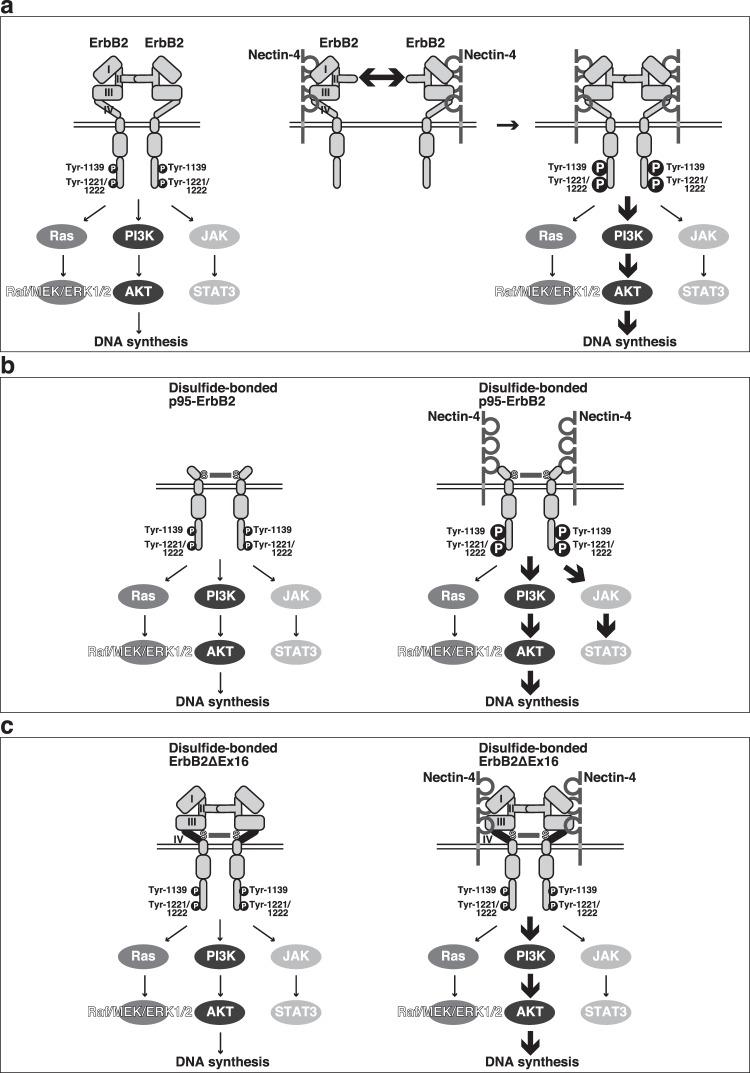
Schematic diagrams for the modes of action of nectin-4 on the signalling pathways of ErbB2 and its trastuzumab-resistant variants for DNA synthesis. (**a**) A mechanism for ErbB2. (**b**) A mechanism for p95-ErbB2. (**c**) A mechanism for ErbB2ΔEx16.

The modes of action of nectin-4 on the signalling pathways of ErbB2 and its trastuzumab-resistant variants for DNA synthesis are schematically demonstrated in Fig. 8a–c. As shown in Fig. 8a, nectin-4 *cis*-interacted with ErbB2 and enhanced its homodimerization and tyrosine-phosphorylation. This interaction selectively induced the activation of the PI3K-AKT signalling pathway for DNA synthesis, but not the Ras-Raf-MEK-ERK1/2 or JAK-STAT3 signalling pathway. As shown in Fig. 8b,c, nectin-4 *cis*-interacted with not only ErbB2 but also its trastuzumab-resistant splice variants, p95-ErbB2 and ErbB2ΔEx16, that are homodimerized through the disulfide bond of two cysteine residues^10,11,16,17^. The *cis*-interaction of nectin-4 with p95-ErbB2 enhanced the phosphorylation of its tyrosine residues at 1139, 1221, and 1222 as well as the threonine-phosphorylation of AKT, similar to that with ErbB2, but additionally enhanced the tyrosine-phosphorylation of STAT3 that was not augmented by the *cis*-interaction of nectin-4 with ErbB2 or ErbB2ΔEx16, as shown in Fig. 8a–c. Although the *cis*-interaction of nectin-4 with ErbB2ΔEx16 did not enhance the phosphorylation of its tyrosine residues at 1139, 1221, or 1222, the threonine-phosphorylation of AKT was enhanced similar to that with ErbB2 as shown in Fig. 8c. These models may provide a clue about a mechanism how the activities of oncogenic cell surface receptors, such as ErbB2 and trastuzumab-resistant splice variants, p95-ErbB2 and ErbB2ΔEx16, are regulated by nectin-4 as a co-receptor.

## Discussion

We showed here for the first time that nectin-4 *cis*-interacted with ErbB2 through the third Ig-like domain of nectin-4 and domain IV of ErbB2 and enhanced its homodimerization and tyrosine-phosphorylation, leading to the selective activation of the PI3K-AKT signalling pathway for DNA synthesis, but not the Ras-Raf-MEK-ERK1/2 or JAK-STAT3 signalling pathway. It was previously shown that the homodimerization of ErbB2 requires its upregulation, although the ligand-dependent homodimerization of ErbB1 or the heterodimerization of ErbB2 with ErbB3 does not require the upregulation of these molecules^1–5^, indicating that the affinity for the interaction between the extended domain II of one ErbB2 molecule and that of another ErbB2 molecule might be lower than the affinity of the interaction between the extended domain II of ErbB1 and that of another ErbB1 molecule as well as the affinity of the interaction between the extended domain II of one ErbB2 molecule and that of another ErbB3 molecule. The *cis*-interaction of nectin-4 with ErbB2 is likely to increase the affinity for the interaction between the extended domain II of one ErbB2 molecule and that of another ErbB2 molecule.

We further showed here that the knockdown of endogenous nectin-4 reduced the tyrosine-phosphorylation of ErbB2 and its activation. However, the level of the tyrosine-phosphorylation of ErbB2 was not always correlated with the expression levels of nectin-4. The exact reason for this discrepancy is not clear, but it was previously shown that the growth factor receptors could dimerize depending on their expression levels and that this dimerization of the receptors enhances their tyrosine-phosphorylation and the activation of their downstream signalling pathways^1–5^. Taken together these previous observations, the present results suggest that nectin-4 is an important co-receptor regulating the tyrosine-phosphorylation level of ErbB2 but may not be the only molecule showing this activity.

It was noted that the *cis*-interaction of nectin-4 with ErbB2 induces the activation of the PI3K-AKT signalling pathway more efficiently than that of the Ras-Raf-MEK-ERK1/2 and JAK-STAT3 signalling pathways. It was previously shown that the Ras-Raf-MEK-ERK signalling pathway is a major mitogenic signalling pathway of ErbB2, but it was subsequently demonstrated that the PI3K-AKT signalling pathway is also a major mitogenic signalling pathway of ErbB2^62,63^. It was more recently reported that upregulated ErbB2 enhances the phosphorylation of tyrosine residues at 1139, 1221, 1222, and 1248 of ErbB2^61^. The mutation of tyrosine residue at 1139 reduces the phosphorylation of AKT, indicating that the tyrosine phosphorylation of this residue on ErbB2 at least leads to the activation of the PI3K-AKT signalling pathway^61^. It remains unclear whether the phosphorylation of other tyrosine residues leads to the activation of the PI3K-AKT signalling pathway. The novel stimulatory mechanism of ErbB2 for its homodimerization and activation by *cis*-interacting with nectin-4, which mainly leads to the selective activation of the PI3K-AKT signalling pathway, may be involved in the enhanced DNA synthesis of cancer cells in which nectin-4 is upregulated. The novel mechanism presented here was consistent with the previous observations that the PI3K-AKT signalling pathway is implicated in the carcinogenic role of nectin-4 in gallbladder, breast, and gastric cancers^50,51,53^.

We further demonstrated here that nectin-4 *cis*-interacted with the trastuzumab-resistant splice variants of ErbB2, p95-ErbB2 and ErbB2ΔEx16, although the trastuzumab-resistance to ErbB2ΔEx16 is controversial^21,22^. It was previously shown that trastuzumab interacts with domain IV of ErbB2, but p95-ErbB2 lacks the trastuzumab-interacting region, which is considered as a mechanism for trastuzumab resistance of p95-ErbB2-expressing breast cancers^13,15,17^. In contrast, the trastuzumab-interacting region is intact in ErbB2ΔEx16, but the efficiency of trastuzumab for its interaction with this region of ErbB2ΔEx16 is less than that of ErbB2^11^. Considering from our data that nectin-4 *cis*-interacted with p95-ErbB2 and ErbB2ΔEx16, the nectin-4-interacting region in domain IV of ErbB2 is likely different from the trastuzumab-interacting region.

We finally showed here that the *cis*-interaction of nectin-4 with p95-ErbB2 enhanced the phosphorylation of its tyrosine residues at 1139, 1221, and 1222 as well as the threonine-phosphorylation of AKT and additionally enhanced the tyrosine-phosphorylation of STAT3. The mechanism for this action of p95-ErbB2 remains unknown, but the phosphorylation of tyrosine residues other than 1139, 1221, and 1222 may be enhanced by this interaction. It was noted that the *cis*-interaction of nectin-4 with ErbB2ΔEx16 enhanced the tyrosine-phosphorylation of AKT, but not the phosphorylation of tyrosine residues at 1139, 1221, and 1222 of ErbB2ΔEx16. The phosphorylation of tyrosine residues other than these residues may be enhanced by this interaction. Thus, nectin-4 *cis*-interacts with ErbB2, p95-ErbB2, and ErbB2ΔEx16, but this interaction may enhance the phosphorylation of different tyrosine residues and/or another unidentified molecule. In contrast to ErbB2, p95-ErbB2 and ErbB2ΔEx16 are homodimerized through the disulfide bond of two cysteine residues^10,11,16,17^. Therefore, it is likely that nectin-4 *cis*-interacts with the dimerized molecule, enhancing their activation, although nectin-4 *cis*-interacts with the monomer of ErbB2 to form a complex, enhancing its dimerization. The molecular mechanisms for the nectin-4-enhanced activation of p95-ErbB2 and ErbB2ΔEx16 remain unknown, but the binding of nectin-4 to domain IV of these molecules may affect the conformational changes of their cytoplasmic regions so that the intermolecular tyrosine-phosphorylation is enhanced.

Enfortumab vedotin, an Ab-drug conjugate targeting nectin-4, has been developed as a highly potent therapeutic agent in multiple preclinical cancer models^58^. This Ab recognizes the first Ig-like domain of nectin-4 and inhibits its *trans*-interaction with nectin-1. Targeting the third Ig-like domain of nectin-4, which *cis*-interacts with ErbB2, might be an alternative strategy for the treatment of trastuzumab-resistant breast cancers expressing p95-ErbB2 and ErbB2ΔEx16, although it was recently reported that trastuzumab is effective for breast cancers expressing ErbB2ΔEx16^21,22^. This novel strategy for the development of therapeutic drugs for trastuzumab-resistant breast cancers may be also effectively used for the screening of chemical compound drugs.

## Methods

### Cell culture and transfection

The method of cell culture was described in Supplementary Methods. Transfection reagents and supplements for cells used in this study were listed in Supplementary Table 1.

### Plasmid construction

The cDNAs used in this study were described in Supplementary Methods.

### Antibodies and reagents

The Abs and reagents used in this study were listed in Supplementary Table 2 and Supplementary Table 1, respectively. Rabbit anti-Necl-1 pAb, rabbit anti-Necl-2 pAb, and rabbit anti-Necl-3 pAb were described previously^64–66^.

### Co-immunoprecipitation assay and Western blotting

The methods of co-immunoprecipitation assay and Western blotting were described in Supplementary Methods. The reagents for the assays were listed in Supplementary Table 1.

### Immunofluorescence microscopy

The methods of confocal image analysis were described in Supplementary Methods. The Abs for the assays were listed in Supplementary Table 2.

### Assay for the phosphorylation of ErbB2, AKT, ERK1/2, and STAT3

T47D or SUM190-PT cells were plated at a density of 3 × 10^4^ cells per square centimetre on dishes and cultured for 2 days. The cells were serum-starved with RPMI-1640 or Ham’s F12 containing 0.5% fatty acid-free BSA in the absence or presence of the inhibitor at 37 °C for 24 h, and then the cells were washed with ice-cold PBS and lysed with the lysis buffer. The lysates were then heated at 80 °C in the SDS sample buffer for 2 min and subjected to SDS-PAGE, followed by Western blotting. The intensity of the bands in Western blotting was calculated using ImageJ 1.48 v 32-bit software.

### siRNA experiments

Silencer select siRNA against human *NECTIN4* and a silencer select siRNA negative control siRNA (AM4611) were purchased (Thermo Fisher Scientific, Waltham, MA, USA). Transfection reagent and siRNA sequences were listed in Supplementary Tables 1 and 3, respectively.

### Assay for DNA synthesis

T47D or SUM190-PT cells were plated at a density of 3 × 10^4^ cells per square centimetre on dishes and cultured at 37 °C for 48 h. The cells were starved of serum, cultured with RPMI1640 or Ham’s F12 containing 0.5% fatty acid-free BSA at 37 °C in the absence or presence of the inhibitors for 24 h and then treated with 10 μM EdU at 37 °C for 12 h. After washout of EdU-containing media, EdU-treated cells were additionally cultured with serum-free medium in the absence or presence of the inhibitors at 37 °C for 12 h and fixed using 4% paraformaldehyde diluted in PBS. The signal for EdU and nuclei in the cells was determined by the Click-iT EdU Alexa Fluor 488 or 647 Imaging Kit (Thermo Fisher Scientific), according to the manufacturer’s protocol. The samples were counted by fluorescence microscopic examination. The images were acquired using a BZ-X710 microscope (KEYENCE CORPORATION, Osaka, Japan) with a CFI Plan Apo λ 20 × /0.75 numerical aperture objective lens (Nikon, Inc., Tokyo, Japan) in 1,920 × 1,440 pixels. The displayed images were applied into maximum signal intensity projection from around 5 confocal images collected at a 2 μm step along the z-axis at room temperature under the control of BZ-X Viewer software (KEYENCE CORPORATION). The images were processed using ImageJ 1.48 v 32-bit software.

### Protein purification and *in vitro* assay for the inhibition of the interaction between nectin-4 and ErbB2

The method of the purification of recombinant proteins was described in Supplementary Methods. Inhibition of the interaction between the extracellular region of nectin-4 and ErbB2 was carried out using recombinant FLAG-tagged nectin-4 Ig1/2/3 and ErbB2-GFP by adding recombinant FLAG-tagged nectin-4 Ig3. ErbB2-GFP was captured on anti-GFP pAb-covalently-conjugated beads by incubating with the lysates of HEK293E cells expressing ErbB2-GFP, and the beads were washed three times with the lysis buffer. The washed beads were resuspended in the lysis buffer containing 5 μM recombinant FLAG-tagged nectin-4 Ig1/2/3 in the presence or absence of 25 μM recombinant FLAG-tagged nectin-4 Ig3 and incubated in a total volume of 20 μl at 4 °C for 16 h with gentle mixing. After ErbB2-GFP were mixed with the recombinant proteins, the beads were washed three times with PBS containing 0.02% Tween-20, and the bound proteins were eluted by heating at 80 °C in the SDS sample buffer for 2 min. The samples were subjected to SDS-PAGE, followed by Western blotting using the indicated Abs.

### Statistical analysis

Statistical significance was analysed using GraphPad Prism 6 software (GraphPad Software Inc., La Jolla, CA, USA) for two-tailed Welch’s t-test for two groups, or one-way analysis of variance followed by a *post hoc* Tukey’s test for comparisons among more than three groups.

## Supplementary information

Supplementary Figures, Tables and Methods

## Data Availability

All of the datasets analyzed and all of the reagents used or generated during this study are available from the corresponding authors on reasonable request.
