# Supplementary material for: Nectin-4 cis-interacts with ErbB2 and its trastuzumab-resistant splice variants, enhancing their activation and DNA synthesis
Source: Sci Rep. 2019 Dec 12;9:18997. doi: 10.1038/s41598-019-55460-9 (PMC6908695; doi:10.1038/s41598-019-55460-9)
Supplement: Supplementary file 1 — Supplementary Figures, Tables and Methods [file 41598_2019_55460_MOESM1_ESM.pdf]

Nectin-4 *cis*-interacts with ErbB2 and its trastuzumab-resistant splice variants, enhancing their activation and DNA synthesis

Shin Kedashiro, Ayumu Sugiura, Kiyohito Mizutani*, and Yoshimi Takai*

From the Division of Pathogenetic Signaling, Department of Biochemistry and Molecular Biology, Kobe University Graduate School of Medicine, 1-5-6 Minatojima-minamimachi, Chuo-ku, Kobe, Hyogo 650-0047, Japan

*Corresponding author:

Yoshimi Takai (ytakai@med.kobe-u.ac.jp)

or

Kiyohito Mizutani (mizutani@med.kobe-u.ac.jp)

Supplementary Figure 1: Specific *cis*-interaction of nectin-4 with ErbB2.

Supplementary Figure 2: The *cis*-interaction of the third immunoglobulin-like domain of nectin-4 with domain IV of ErbB2.

Supplementary Figure 3: Inhibition of the *cis*-interaction of nectin-4 with ErbB2 by recombinant protein of the third immunoglobulin-like domain of nectin-4.

Supplementary Figure 4: The full-length blots of Figure 2.

Supplementary Figure 5: The full-length blots of Figure 3.

Supplementary Figure 6: The full-length blots of Figure 4a and Figure 4b.

Supplementary Figure 7: The full-length blots of Figure 4c.

Supplementary Figure 8: The full-length blots of Figure 6.

Supplementary Figure 9: The full-length blots of Figure 7.

Supplementary Figure 10: The full-length blots of Supplementary Figure 1.

Supplementary Figure 11: The full-length blots of Supplementary Figure 2.

Supplementary Figure 12: The full-length blots of Supplementary Figure 3.

Supplementary Table 1: The list of the reagents used in this study.

Supplementary Table 2: The list of the antibodies used in this study.

Supplementary Table 3: The list of the siRNA sequences used in this study.

Supplementary Methods

Supplementary Figure 1

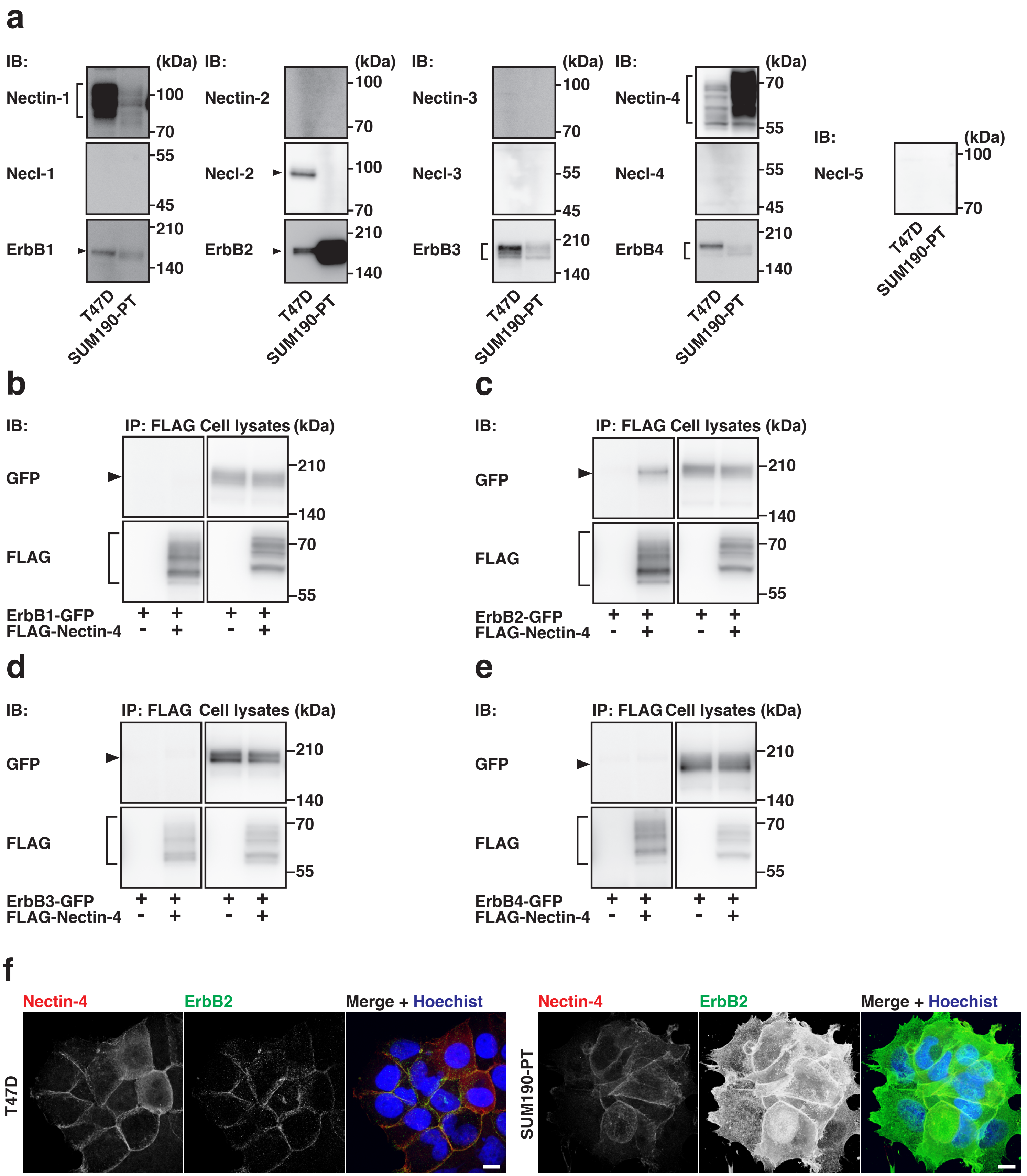

Supplementary Figure 1. Specific *cis*-interaction of nectin-4 with ErbB2. **a** Expression of nectin and ErbB family members. Lysates of T47D and SUM190-PT cells (20 µg of each protein) were subjected to Western blotting using the indicated Abs. **b–e** Specific *cis*-interaction of nectin-4 with ErbB2. HEK293E cells were co-transfected with various combinations of the indicated plasmids and cultured in suspension. FLAG-tagged nectin-4 (FLAG-Nectin-4) was immunoprecipitated using anti-FLAG mAb, and samples were subjected to Western blotting using the indicated Abs. **f** Localization of nectin-4 and ErbB2 in T47D and SUM190-PT cells. Each of the cells were stained with the indicated Abs. Arrowheads and square brackets indicate each of the proteins. The displayed blots were cropped, and the full-length blots are shown in Supplementary Figure 10. Scale bars, 10 µm. IB, immunoblotting; IP, immunoprecipitation. Representative results from three independent experiments are shown.

Supplementary Figure 2

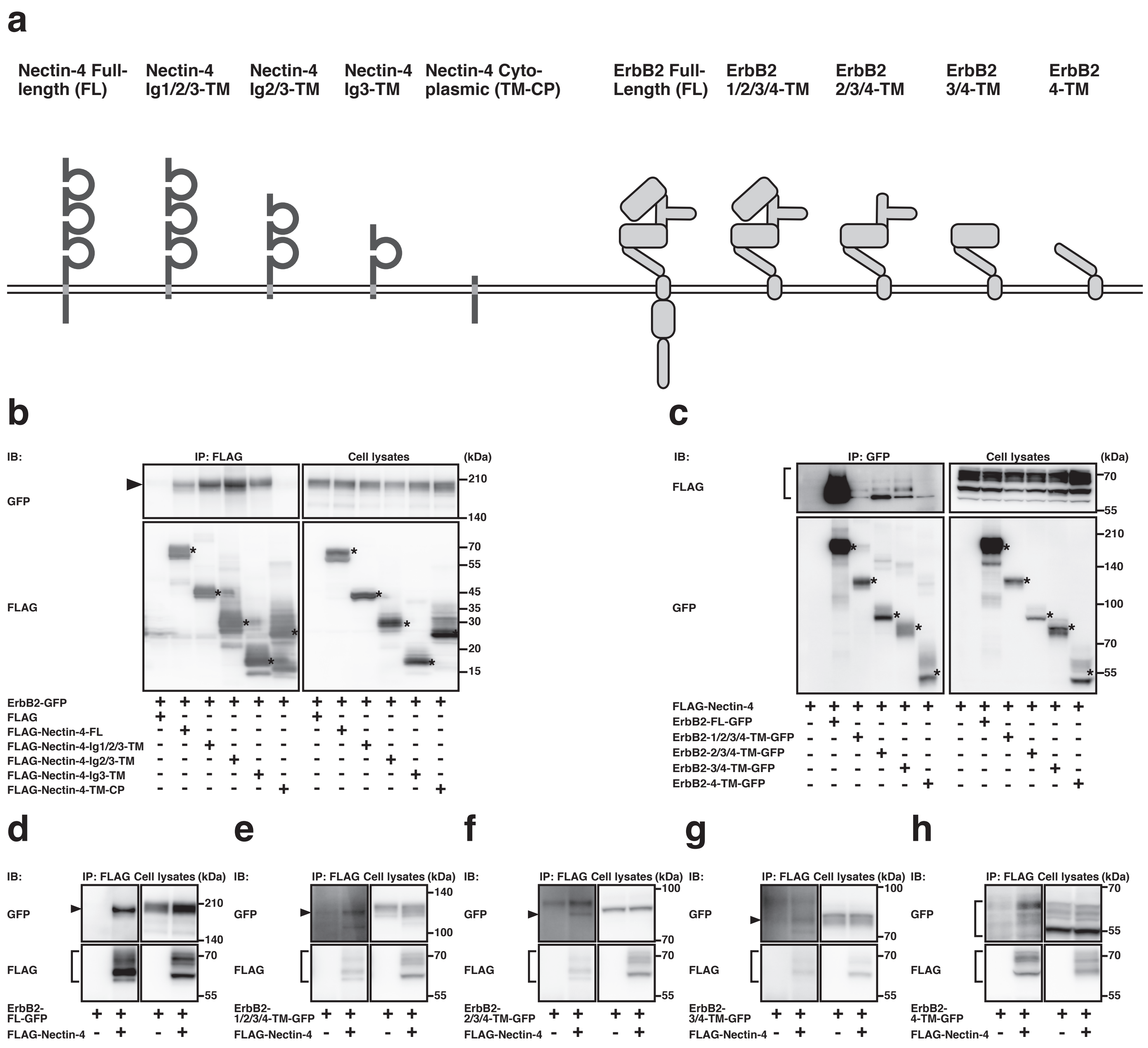

Supplementary Figure 2. The *cis*-interaction of the third immunoglobulin-like domain of nectin-4 with domain IV of ErbB2. a Schematic of nectin-4 mutants and ErbB2 mutants. **b** Identification of the interaction domain of nectin-4 with ErbB2. HEK293E cells were co-transfected with various combinations of the indicated plasmids and cultured in suspension. FLAG-tagged nectin-4 (FLAG-Nectin-4) and its immunoglobulin (Ig)-like domain deleated mutants were immunoprecipitated using the anti-FLAG mAb, and the samples were subjected to Western blotting using the indicated Abs. **c–h** Identification of the interaction domain of ErbB2 with nectin-4. HEK293E cells were co-transfected with various combinations of the indicated plasmids and cultured in suspension. GFP-tagged ErbB2 mutants were immunoprecipitated using the anti-GFP pAb (**c**). FLAG-Nectin-4 was immunoprecipitated using the anti-FLAG mAb (**d–h**). The samples were subjected to Western blotting using the indicated Abs. Arrowheads and square brackets indicate each of the protein. The displayed blots were cropped, and the full-length blots are shown in Supplementary Figure 11. Asterisks, nectin-4 mutants or ErbB2 mutants; IB, immunoblotting; IP, immunoprecipitation. Representative results from three independent experiments are shown.

Supplementary Figure 3

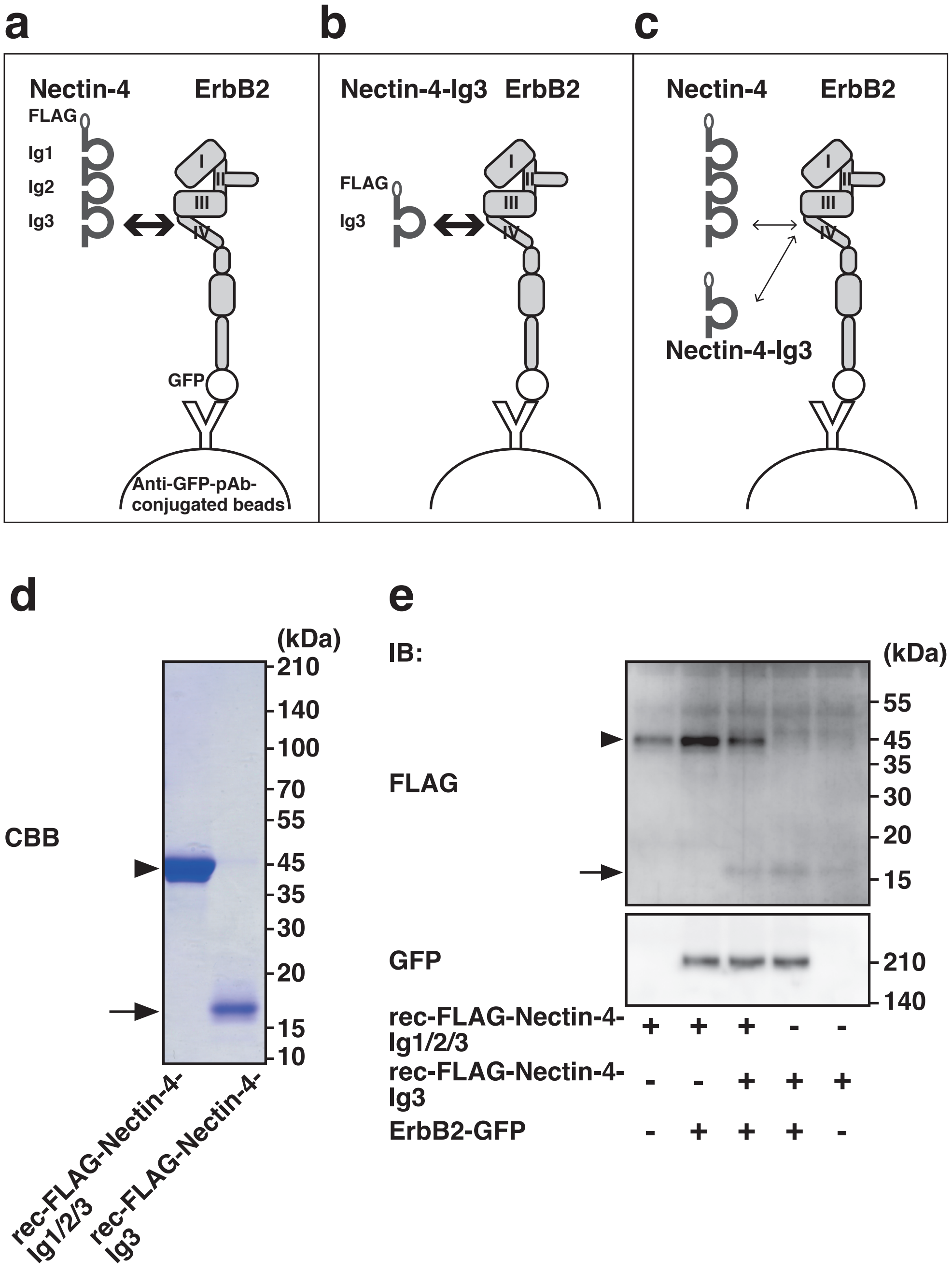

Supplementary Figure 3. Inhibition of the *cis*-interaction of nectin-4 with ErbB2 by recombinant protein of the third immunoglobulin-like domain of nectin-4. a–c Schematic diagram of the experimental design. **d** Purification of FLAG-tagged recombinant proteins of nectin-4. Recombinant FLAG-tagged extracellular region of nectin-4 (rec-FLAG-Nectin-4-Ig1/2/3) and recombinant FLAG-tagged third immunoglobulin (Ig)-like domain of nectin-4 (rec-FLAG-Nectin-4-Ig3) were prepared from HEK293E cells. The purified proteins (100 pmol of protein each) were analysed by SDS-PAGE with Coomassie brilliant blue (CBB) staining. **e** Inhibition of the *cis*-interaction of nectin-4 with ErbB2 by recombinant protein of the third Ig-like domain of nectin-4. ErbB2-GFP was immobilized on anti-GFP-Ab-conjugated beads. The beads were resuspended in PBS containing 5 μM rec-FLAG-Nectin-4-Ig1/2/3 in the presence or absence of 25 μM rec-FLAG-Nectin-4-Ig3 and incubated in a total volume of 20 μl at 4°C for 16 h with gentle mixing. The beads were precipitated, and the samples were subjected to Western blotting using the indicated Abs. Arrowheads or arrows indicate the bands of rec-FLAG-nectin-4-Ig1/2/3 or rec-FLAG-Nectin-4-Ig3, respectively. The displayed blots were cropped, and the full-length blots are shown in Supplementary Figure 12. IB, immunoblotting. Representative results from three independent experiments are shown.

Supplementary Figure 4

a

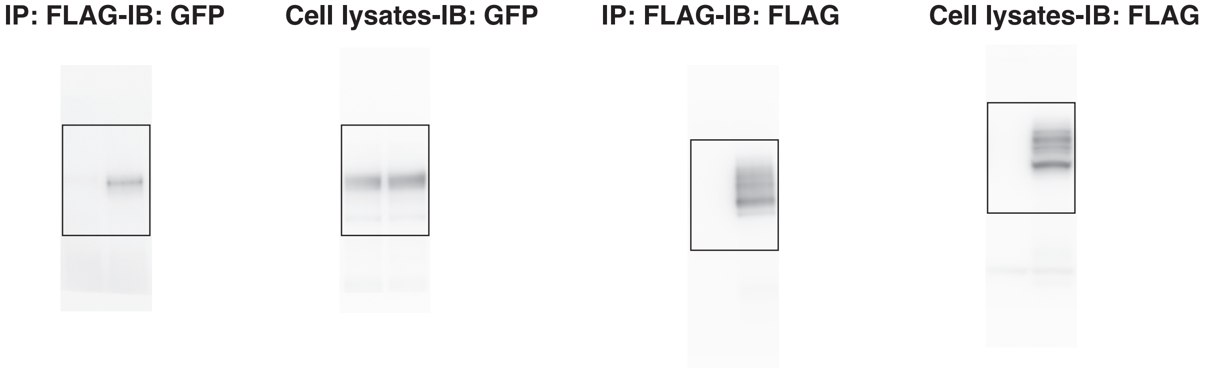

b

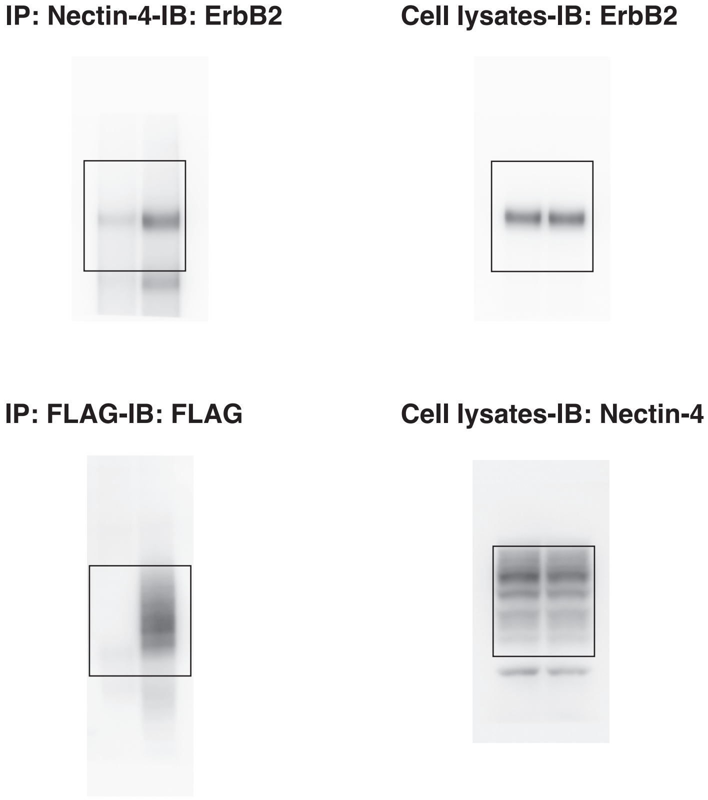

Supplementary Figure 4. Full-length blots of Figure 2. a-b full-length blots of Figure 2a-b, respectively. Boxed regions were cropped and shown as the figure.

Supplementary Figure 5

a

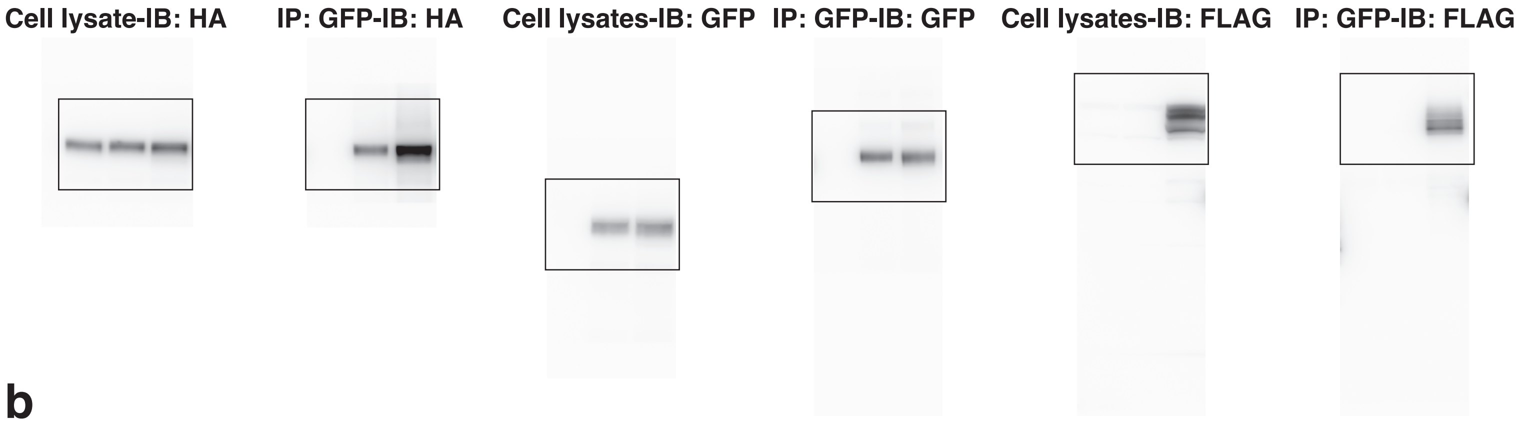

b

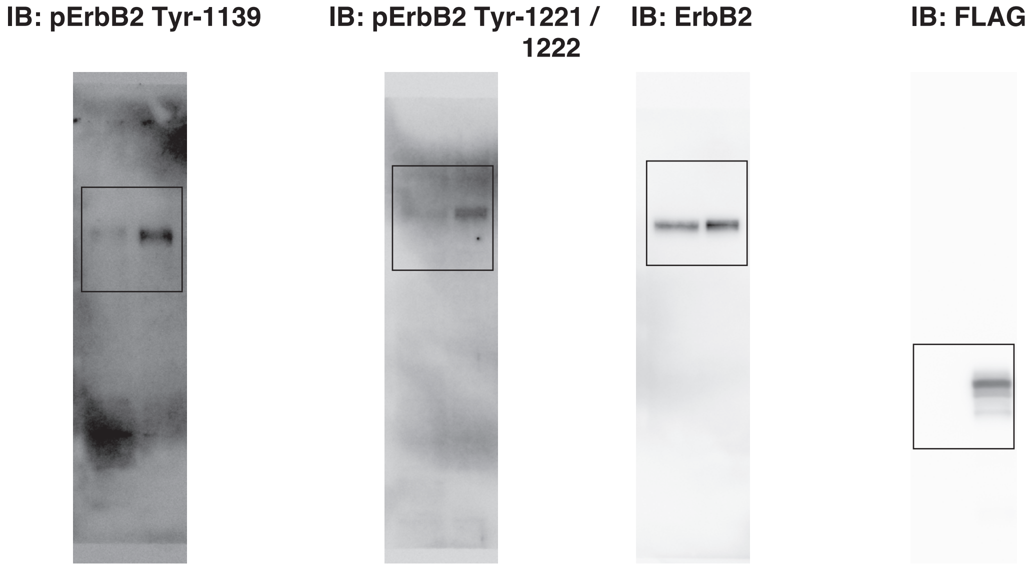

c

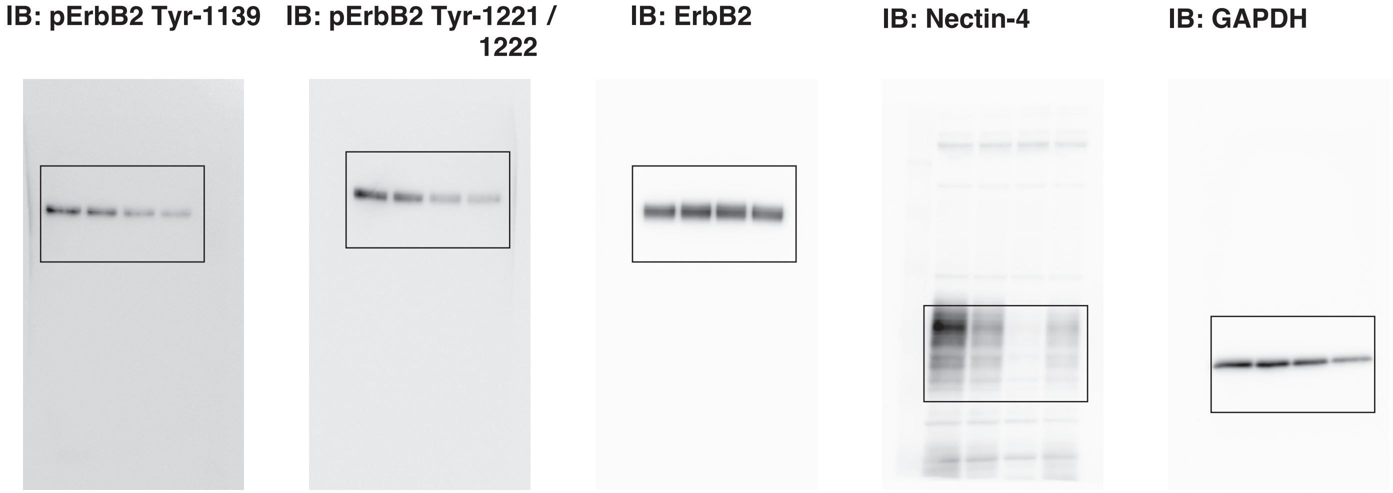

Supplementary Figure 5. Full-length blots of Figure 3. a-c full-length blots of Figure 3a-c, respectively. Boxed regions were cropped and shown as the figure.

Supplementary Figure 6

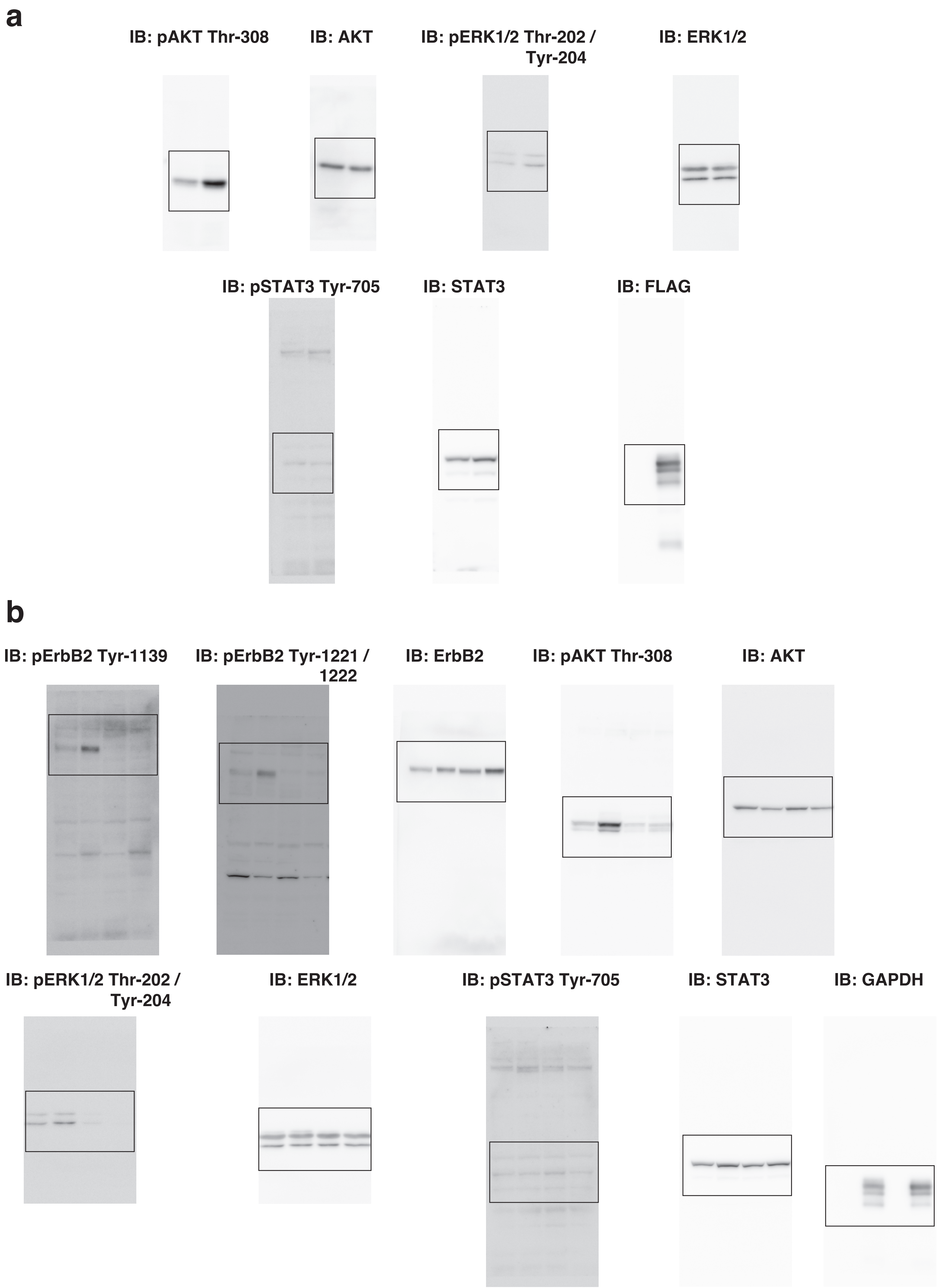

Supplementary Figure 6. Full-length blots of Figure 4a-b. a-b full-length blots of Figure 4a-b, respectively. Boxed regions were cropped and shown as the figure.

Supplementary Figure 7

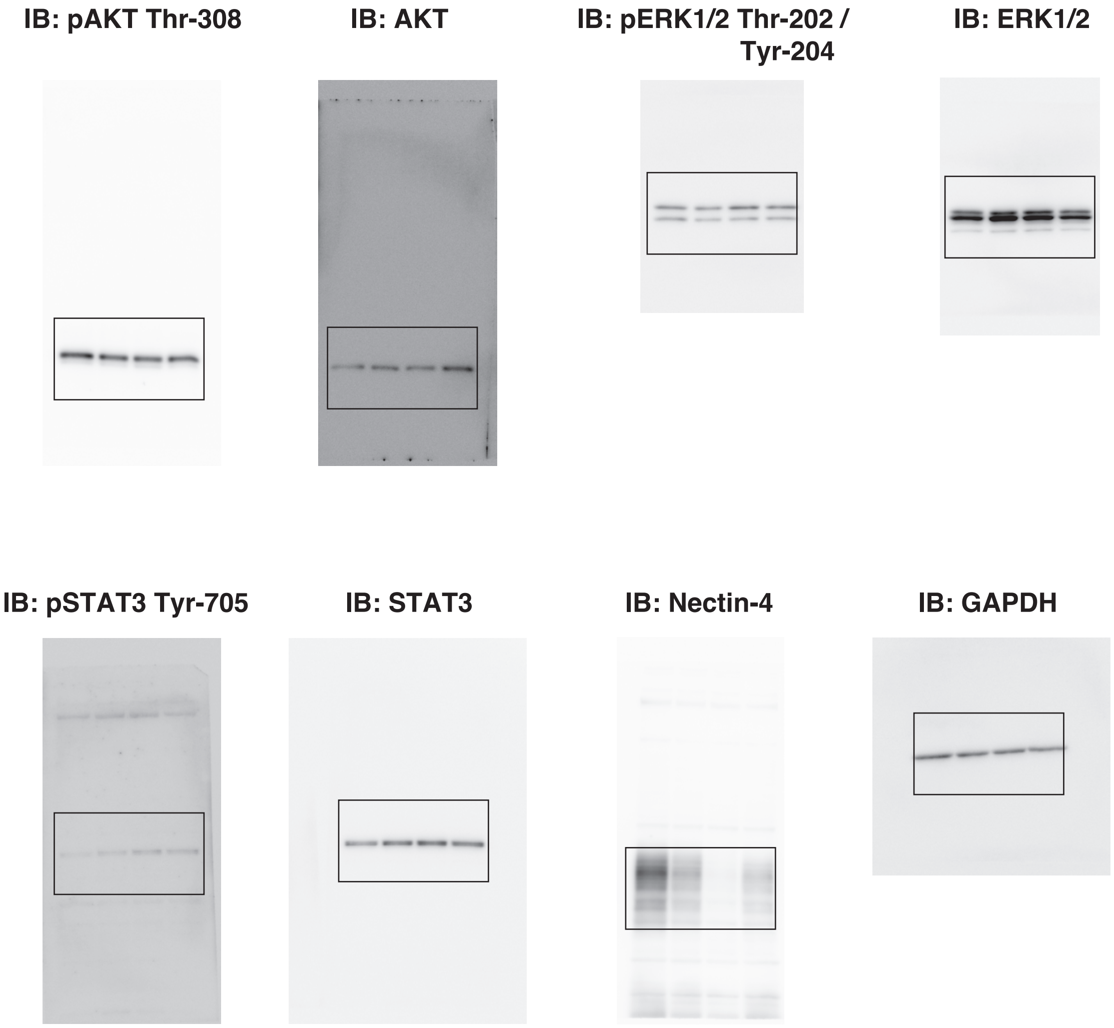

Supplementary Figure 7. Full-length blots of Figure 4c. full-length blots of Figure 4c in this manuscript. Boxed regions were cropped and shown as the figure.

Supplementary Figure 8

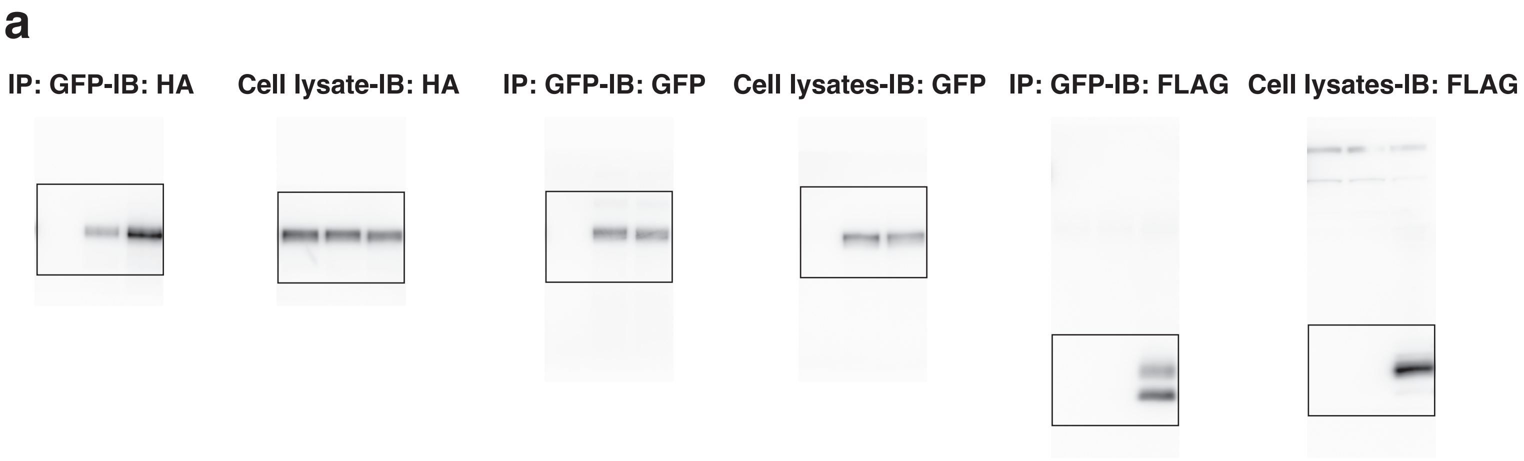

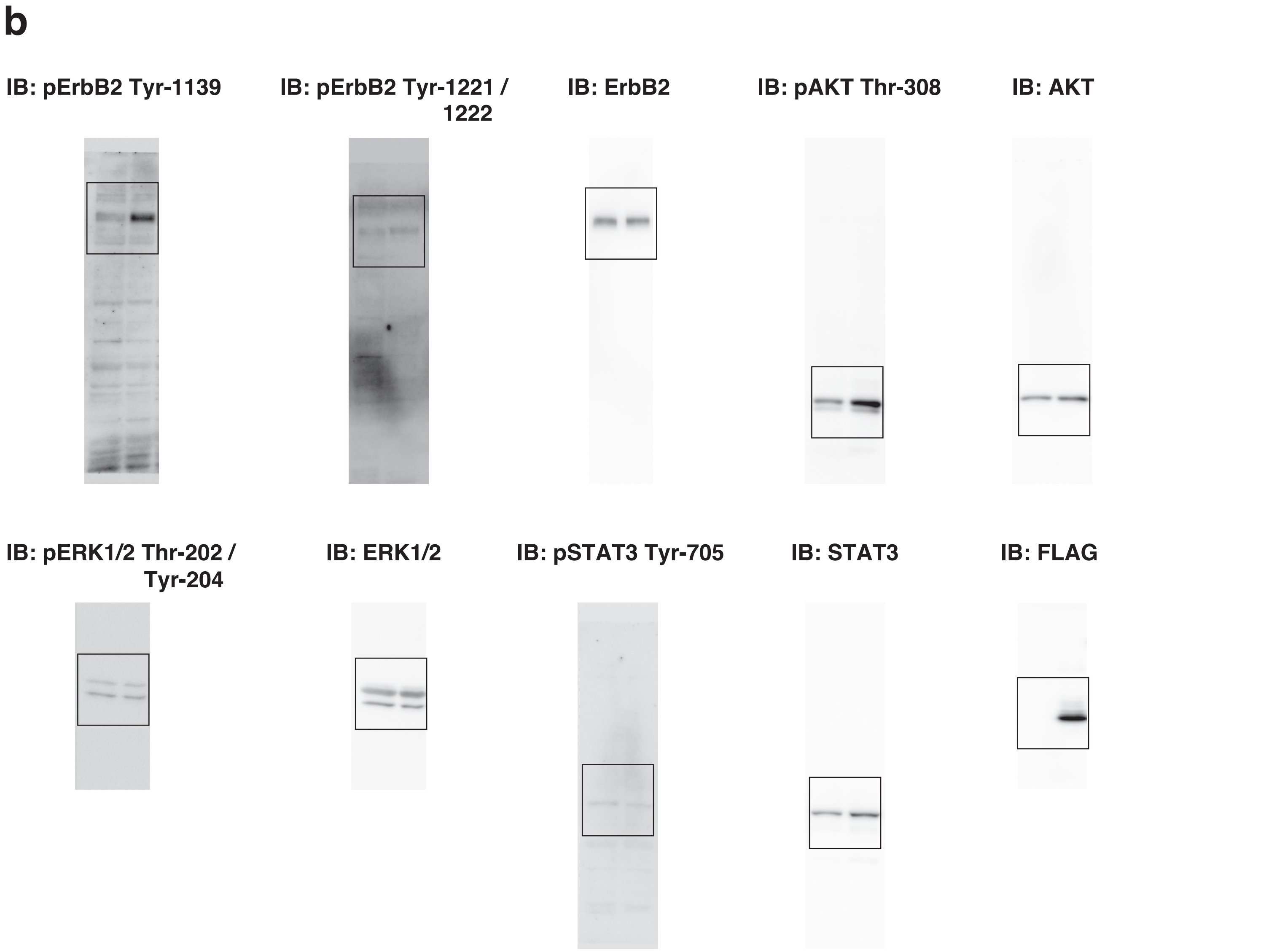

Supplementary Figure 8. Full-length blots of Figure 6. a-b full-length blots of Figure 6a-b, respectively. Boxed regions were cropped and shown as the figure.

Supplementary Figure 9

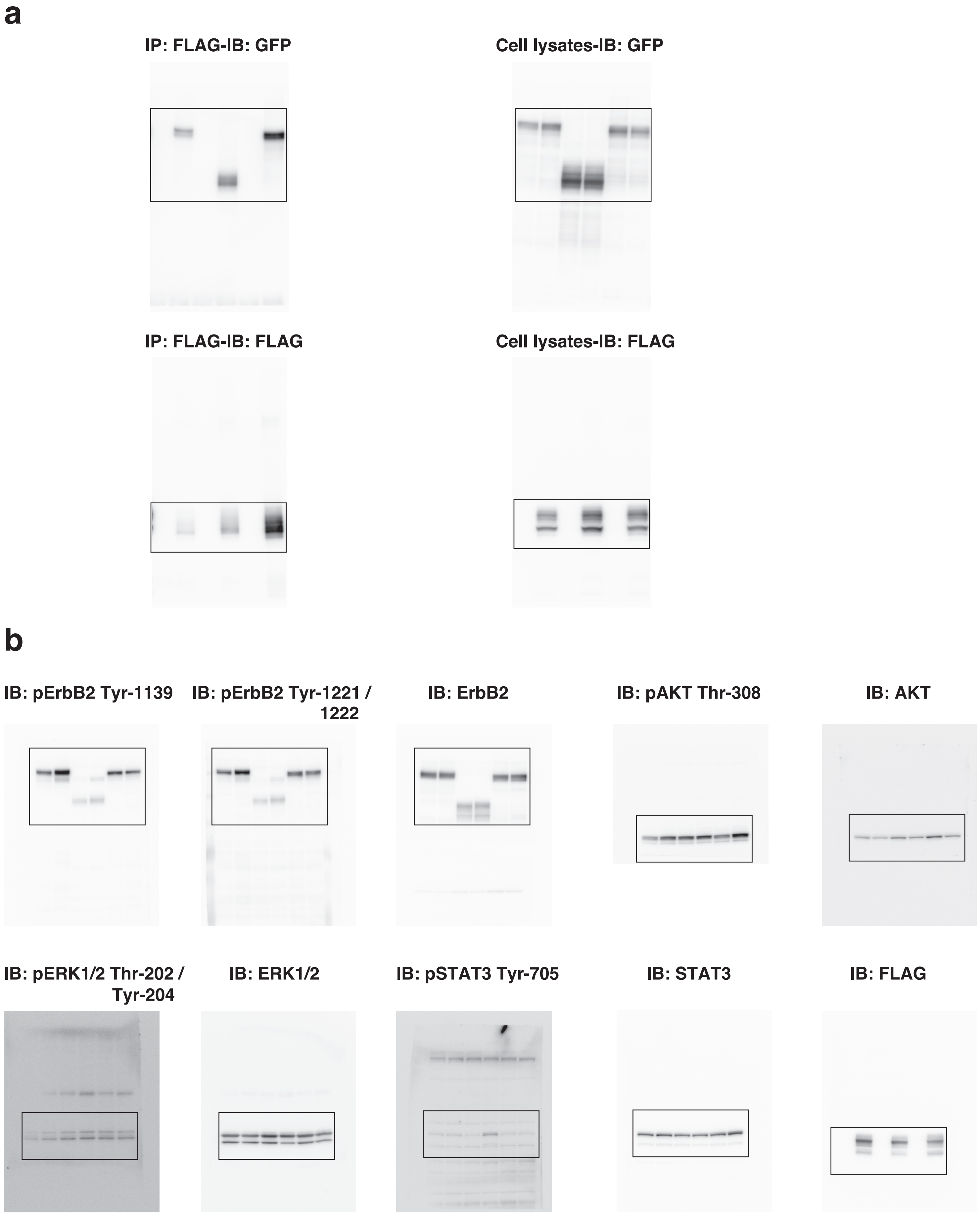

Supplementary Figure 9. Full-length blots of Figure 7. a full-length blots of Figure 7b in this manuscript. Boxed regions were cropped and shown as the figure. **b** full-length blots of Figure 7c in this manuscript. Boxed regions were cropped and shown as the figure.

Supplementary Figure 10

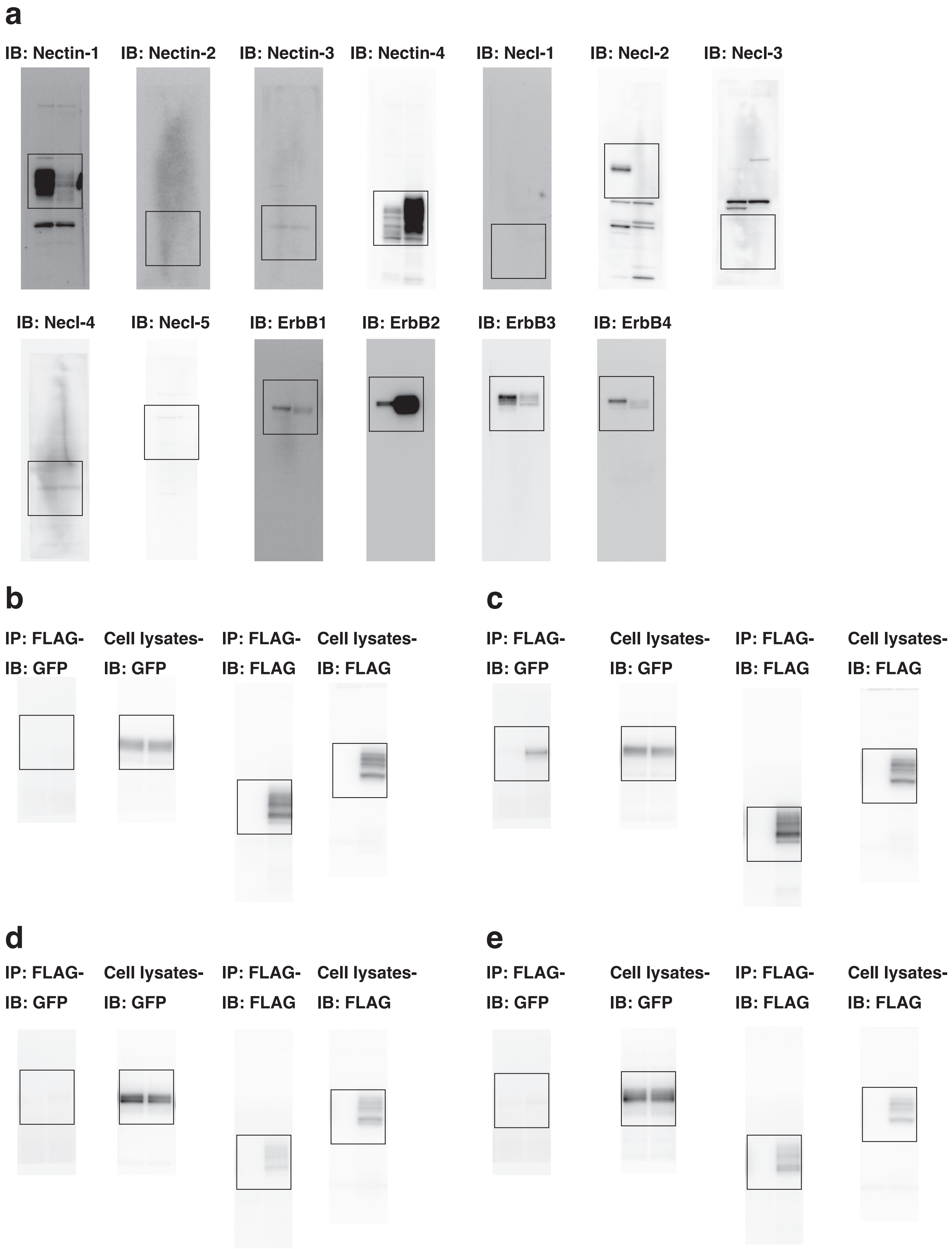

Supplementary Figure 9. Full-length blots of Supplementary Figure 1. a-e full-length blots of Suppmentary Figure 1a-e, respectively. Boxed regions were cropped and shown as the figure.

Supplementary Figure 11

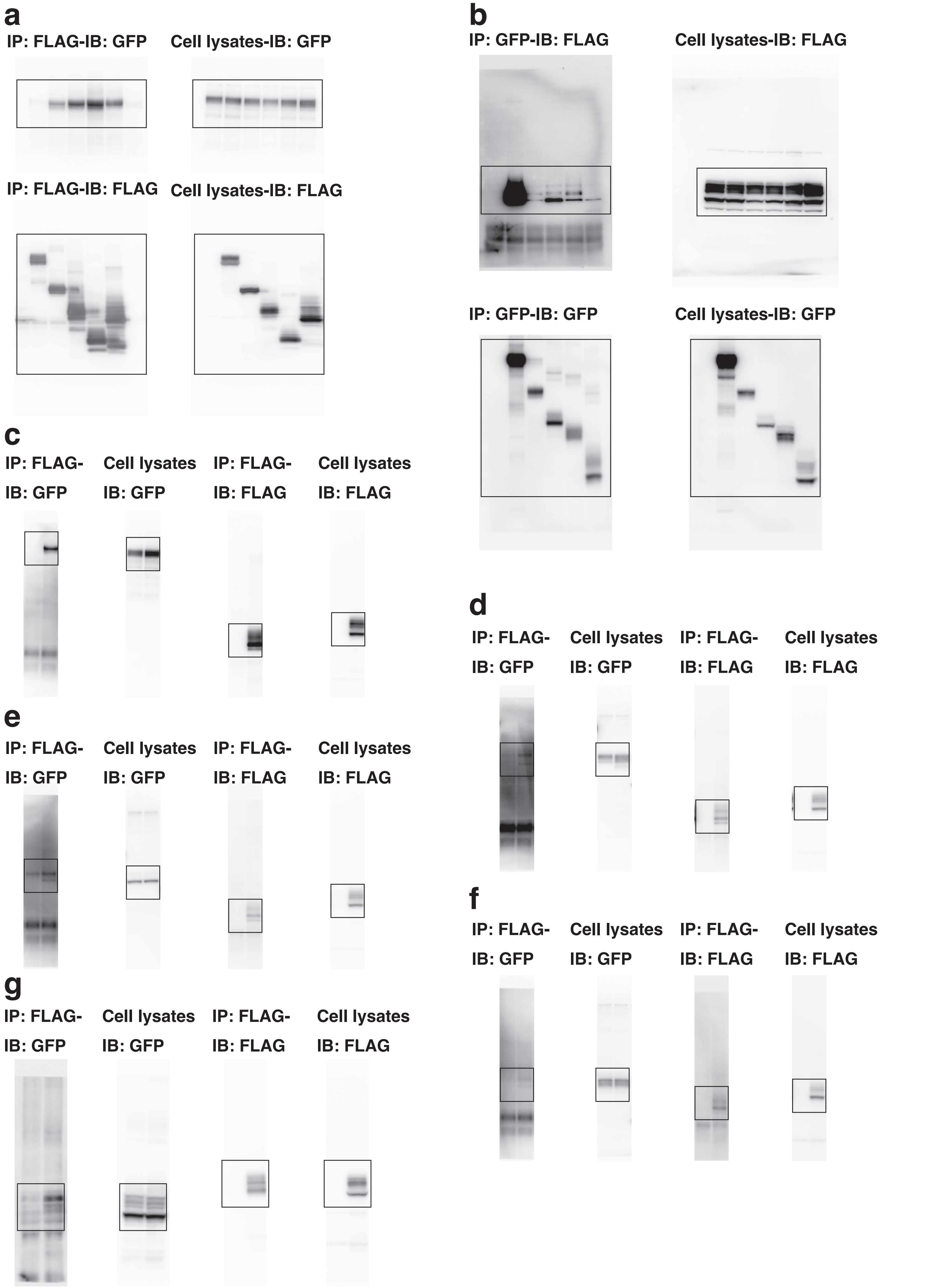

Supplementary Figure 11. Full-length blots of Supplementary Figure 2. a-g full-length blots of Supplementary Figure 2b-h. Each of the full-length blots are compatible with Supplementary Figure 2, respectively in a way of off-by-one. Boxed regions were cropped and shown as the figure.

Supplementary Figure 12

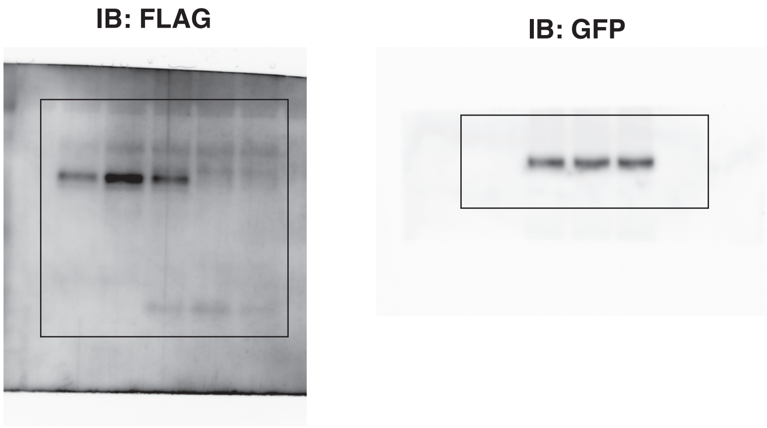

Supplementary Figure 12. Full-length blots of Supplementary Figure 3. full-length blots of Supplementary Figure 3e in this manuscript. Boxed regions were cropped and shown as the figure.

Supplementary Table 1

Reagent	Experiment	Supplier	Address	Catalogue number
**Cell culture and transfection**				
Amaxa Cell Line Nucleofector Kit V	Transfection for T47D	Lonza	Allendale, NJ, USA	VCA-1003
Apo-transferrin	Supplements for SUM190-PT	Sigma-Aldrich	St. Louis, MO, USA	T2252
Blasticidin	Stable expression for ErbB2	Wako Pure Chemical Corporation	Osaka, Japan	029-18701
Ethanolamine	Supplements for SUM190-PT	Sigma-Aldrich	St. Louis, MO, USA	E0135
Fatty acid-free BSA	Cell culture	Sigma-Aldrich	St. Louis, MO, USA	A8806
HEPES	Supplements for SUM190-PT	Nacalai Tesque	Kyoto, Japan	15639-84
Hydrocortisone	Supplements for SUM190-PT	Sigma-Aldrich	St. Louis, MO, USA	H4001
Insulin	Cell culture	Sigma-Aldrich	St. Louis, MO, USA	I9278
Irbinitinib	DNA synthesis and WB	Selleck Chemicals	Houston, TX, USA	S8362
Lipofectamine 3000	Transfection for HEK293E	Thermo Fisher Scientific	Waltham, MA, USA	L3000075
LY294002	DNA synthesis	Calbiochem	Billerica, MA, USA	440202
Puromycin	Stable expression for Nectin-4	Wako Pure Chemical Corporation	Osaka, Jpan	166-23153
Ruxolitinib	DNA synthesis	Selleck Chemicals	Houston, TX, USA	S1378
Serum-free media	Protein purification	SAFC Biosciences Inc.	Lenexa, KS, USA	24571C
Sodium selenite	Supplements for SUM190-PT	Sigma-Aldrich	St. Louis, MO, USA	S9133
Triiodo thyronine	Supplements for SUM190-PT	Sigma-Aldrich	St. Louis, MO, USA	T5516
U0126	DNA synthesis	Calbiochem	Billerica, MA, USA	662005
Wortmannin	DNA synthesis	Calbiochem	Billerica, MA, USA	681675
Xfect MicroRNA Transfection Reagent	Transfection for siRNA	Takara Bio	Kusatsu, Japan	631435
**Co-immunoprecipitation and WB**				
Accutase	Co-immunoprecipitation	Nacalai Tesque	Kyoto, Japan	12679-54
Protease inhibitor cocktail (cOmplete, EDTA-free)	Co-immunoprecipitation and WB	Roche Diagnostics GmbH	Mannheim, Germany	11836170001
Phosphatase Inhibitor Cocktail 2	Co-immunoprecipitation and WB	Sigma-Aldrich	St. Louis, MO, USA	P5726
Phosphatase Inhibitor Cocktail 3	Co-immunoprecipitation and WB	Sigma-Aldrich	St. Louis, MO, USA	P0044
Dynabeads protein A	Co-immunoprecipitation	Thermo Fisher Scientific	Waltham, MA, USA	DB10002
Dynabeads protein G	Co-immunoprecipitation	Thermo Fisher Scientific	Waltham, MA, USA	DB10004
Block Ace	WB	KAC	Kyoto, Japan	UKB80
Can Get Signal Solution 1	WB	Toyobo	Osaka, Japan	NKB-201
Can Get Signal Solution 2	WB	Toyobo	Osaka, Japan	NKB-301
Immobilon Western Chemiluminescent HRP Substrate	WB	Merck Millipore	Billerica, MA, USA	WBKLS0500
**Protein purification**				
DDDDK-antibody-conjugated beads	Protein purification	MBL International	Nagoya, Japan	3328
DDDDK-tag peptide	Protein purification	MBL International	Nagoya, Japan	3325-205

Supplementary Table 1. The list of the reagents used in this study. WB, western blotting; HRP, horseradish peroxidase.

Supplementary Table 2

Antibody	Experiment	Dilution	Buffer for IB or IF	Host	Clone	Clone number	Supplier	Address	Catalogue number
**Primary antibody**									
AKT	IB	1 / 1000	Block Ace	Rabbit	Polyclone	-	Cell Signaling Technology	Danvers, MA, USA	9272
Phospho-AKT (Thr308)	IB	1 / 5000	Can Get	Rabbit	Polyclone	-	Cell Signaling Technology	Danvers, MA, USA	9275
DDDDK	IF	1 / 1000	PBS with 3% BSA	Mouse	Monoclone	FLA-1	MBL International	Nagoya, Japan	M185
DDDDK-HRP	IB	1 / 10000	Can Get	Mouse	Monoclone	FLA-1	MBL International	Nagoya, Japan	M185-7
ErbB1	IB	1 / 500	Block Ace	Mouse	Monoclone	6F1	MBL International	Nagoya, Japan	MI-12-1
ErbB2	IB, IF	IB: 1 / 1000, IF: 1 / 100	Block Ace, PBS with 3% BSA	Rabbit	Monoclone	29D8	Cell Signaling Technology	Danvers, MA, USA	2165
ErbB2-HRP	IB	1 / 5000	Can Get	Rabbit	Monoclone	29D8	Cell Signaling Technology	Danvers, MA, USA	60388S
Phospho-ErbB2 (Tyr1139)	IB	1 / 5000	Can Get	Rabbit	Monoclone	EP1046Y	Abcam	Cambridge, UK	ab53290
Phospho-ErbB2 (Tyr1221/1222)	IB	1 / 5000	Can Get	Rabbit	Monoclone	6B12	Cell Signaling Technology	Danvers, MA, USA	2243
ErbB3	IB	1 / 1000	Block Ace	Rabbit	Monoclone	D22C5	Cell Signaling Technology	Danvers, MA, USA	12708
ErbB4	IB	1 / 1000	Block Ace	Rabbit	Monoclone	111B2	Cell Signaling Technology	Danvers, MA, USA	4795
ERK1/2	IB	1 / 1000	Block Ace	Rabbit	Polyclone	-	Cell Signaling Technology	Danvers, MA, USA	9102
Phospho-ERK1/2 (Thr202/Tyr204)	IB	1 / 2000	Block Ace	Rabbit	Monoclone	D13.14.4E	Cell Signaling Technology	Danvers, MA, USA	4370
FLAG	IB	1 / 1000	Block Ace	Mouse	Monoclone	M2	Sigma-Aldrich	St. Louis, MO, USA	F3165
FLAG	IB, IP	IB: 1 / 1000, IP: 1 / 100	Block Ace	Rabbit	Polyclone	-	Sigma-Aldrich	St. Louis, MO, USA	F7425
GAPDH	IB	1 / 1000	Block Ace	Rabbit	Monoclone	14C10	Cell Signaling Technology	Danvers, MA, USA	2118
GFP	IB, IP	IB: 1 / 1000, IP: 1 / 100	Block Ace	Rabbit	Polyclone	-	MBL International	Nagoya, Japan	598
HA	IB	1 / 1000	Block Ace	Mouse	Monoclone	16B12	BioLegend	San Diego, CA, USA	MMS-101P
Nectin-1	IB	1 / 500	Block Ace	Rabbit	Polyclone	-	Santa Cruz Biotechnology Inc.	Santa Cruz, CA, USA	SC-28639
Nectin-2	IB	1 / 500	Block Ace	Rat	Monoclone	502-57	MBL International	Nagoya, Japan	D083-3
Nectin-3	IB	1 / 500	Block Ace	Rabbit	Polyclone	-	Santa Cruz Biotechnology Inc.	Santa Cruz, CA, USA	SC-14806
Nectin-4	IB, IF, IP	IB: 1 / 1000, IF: 1 / 100, IP: 1 / 50	Block Ace, PBS with 3% BSA	Goat	Polyclone	-	R&D Systems, Inc.	Minneapolis, MN, USA	AF2659
Necl-1	IB	1 / 500	Block Ace	Rabbit	Polyclone	-	-	-	Reffered in Materials and methods
Necl-2	IB	1 / 500	Block Ace	Rabbit	Polyclone	-	-	-	Reffered in Materials and methods
Necl-3	IB	1 / 500	Block Ace	Rabbit	Polyclone	-	-	-	Reffered in Materials and methods
Necl-4	IB	1 / 1000	Block Ace	Mouse	Monoclone	N244/5	NeuroMab	Davis, CA, USA	75-247
Necl-5	IB	1 / 1000	Block Ace	Mouse	Monoclone	300907	R&D Systems, Inc.	Minneapolis, MN, USA	MAB25301
STAT3	IB	1 / 1000	Block Ace	Mouse	Monoclone	124H6	Cell Signaling Technology	Danvers, MA, USA	9139
Phospho-STAT3 (Tyr705)	IB	1 / 5000	Can Get	Rabbit	Monoclone	D3A7	Cell Signaling Technology	Danvers, MA, USA	9145
**Secondary antibody**									
Mouse IgG-HRP	IB	1 / 10000	Block Ace	Sheep	Polyclone	-	GE Healthcare	Little Chalfont, UK	NA931
Rabbit IgG-HRP	IB	1 / 10000	Block Ace or Can Get	Donkey	Polyclone	-	GE Healthcare	Little Chalfont, UK	NA934
Rat IgG-HRP	IB	1 / 10000	Block Ace	Goat	Polyclone	-	GE Healthcare	Little Chalfont, UK	NA935
Goat IgG-HRP	IB	1 / 10000	Block Ace	Donkey	Polyclone	-	Santa Cruz Biotechnology Inc.	Santa Cruz, CA, USA	sc-2020
Rabbit IgG-Alexa Fluor 488	IF	1 / 200	PBS with 3% BSA	Donkey	Polyclone	-	Abcam	Cambridge, UK	ab150130
Goat IgG-Alexa Fluor 555	IF	1 / 200	PBS with 3% BSA	Donkey	Polyclone	-	Abcam	Cambridge, UK	ab150073
Mouse IgG-Alexa Fluor 555	IF	1 / 2000	PBS with 3% BSA	Goat	Polyclone	-	Thermo Fisher Scientific	Waltham, MA, USA	A21424

Supplementary Table 2. The list of antibodies used in this study. IB, immunoblotting; IF, immunofluorescence; IP, immunoprecipitation. HRP, horseradish peroxidase; Can Get, Canget Signal Solution.

Supplementary Table 3

siRNA	Sequence	Supplier	address	catalogue number
Nectin-4 #1	5'-GGCCUGCUGCAUGUACAUA-3'	Thermo Fisher Scientific	Waltham, MA, USA	s37689
Nectin-4 #2	5'-ACUGACUGCUUGACCUUUA-3'	Thermo Fisher Scientific	Waltham, MA, USA	s37690
Nectin-4 #3	5'-AGAUGACCCAGAAAUAUGA-3'	Thermo Fisher Scientific	Waltham, MA, USA	s37691

Supplementary Table 3. The list of siRNA sequences used in this study.

Supplementary Methods

Cell culture and transfection

HEK293E cells were maintained in Dulbecco's modified Eagle's medium supplemented with 10% fetal bovine serum (FBS) at 37°C in 5% CO_2. Human mammary ductal carcinoma T47D cells were maintained in RPMI-1640 supplemented with 10% FBS and 10 μg/ml insulin and cultured at 37°C in 5% CO_2. Human basal breast epithelial carcinoma SUM190-PT cells were purchased from Asterand Bioscience (Detroit, MI, USA) and were maintained in Ham's F-12 supplemented with 0.1% fatty acid-free BSA, 5 mM ethanolamine, 10 mM HEPES at pH 7.5, 1 μg/ml hydrocortisone, 5 μg/ml insulin, 50 nM sodium selenite, 5 μg/ml apo-transferrin, 10 nM triiodo thyronine, and 2% FBS, and cultured at 37°C in 5% CO_2. For the transient expression of various constructs, Lipofectamine 3000 was used for HEK293E cells according to the manufacturer's protocol. For stable expression of nectin-4 and ErbB2 in T47D cells, electroporation was carried out using Amaxa Cell Line Nucleofector Kit V according to the manufacturer's protocol.

Plasmid construction

The following cDNAs were kindly provided: human *ERBB1* and *ERBB2* from Dr. T. Yamamoto (Okinawa Institute of Science and Technology Graduate University, Japan) and human *ERBB3* and *ERBB4* from Dr. S. Higashiyama (Ehime University, Japan). To obtain p95-ErbB2, ErbB2ΔEx16, and various mutants of the extracellular region of ErbB2, in which the cytoplasmic region (deletion of the signalling peptide and the

cytoplasmic region, corresponding to amino acids (aa) 1–22 and 720–1255, respectively), the cytoplasmic region and domain I (deletion of the signalling peptide, domain I, and the cytoplasmic region, corresponding to aa 1–22, 23–193, and 720–1255, respectively), the cytoplasmic region and domains I and II (deletion of the signalling peptide, domains I and II, and the cytoplasmic region, corresponding to aa 1–22, 23–313, and 720–1255, respectively), or the cytoplasmic region and domains I, II, and III (deletion of the signalling peptide, domains I, II, and III, and the cytoplasmic region, corresponding to aa 1–22, 23–509, and 720–1255, respectively) were deleted, each cDNA fragment was amplified by PCR and inserted into pEGFP-N3 vector (Clontech, Mountain View, CA, USA). Preprotrypsin signal peptide was inserted into the plasmids of all the ErbB2 mutants at the N-terminus. pFLAG-CMV1-Nectin-4 and the various mutants of nectin-4 were prepared as described [44]. For the stable expression of FLAG-tagged nectin-4 and FLAG-tagged nectin-4-Ig3-TM, the cDNA was introduced into the pCAGI-Puro vector. For the stable expression of GFP-tagged ErbB2, p95-ErbB2, and ErbB2ΔEx16, the cDNA fragments were amplified by PCR and introduced into pEF-BOS-IRES-blasticidin vector that was constructed by insertion of IRES-blasticidin sequence obtained from pCX4-BSR vector into pEF-BOS vector[67]. For the stable expression of recombinant FLAG-tagged nectin-4 Ig1/2/3 (deletion of the signalling peptide and the cytoplasmic region, corresponding to aa 1–29 and 343–508 respectively) and FLAG-tagged nectin-4-Ig3 (deletion of the signalling peptide, the first and second Ig-like domains and the cytoplasmic region, corresponding to aa 1–29, 30–239, and 343–508, respectively), the cDNA fragments were introduced into the

pCAGI-Puro vector.

Co-immunoprecipitation assay and Western blotting

SUM190-PT or HEK293E cells co-transfected with various combinations of the indicated plasmids were cultured at 37°C for 48 h. The cells were washed with PBS and detached with Accutase. The detached cells were additionally cultured in suspension with Ham’s F12 containing the supplements or Dulbecco’s modified Eagle’s medium containing 10% FBS for 1 h. The cells were collected by centrifugation, washed with ice-cold PBS, and lysed with a lysis buffer (20 mM Tris-HCl at pH 7.5, 1% Nonidet P-40, 10% glycerol, 150 mM NaCl, 1 mM DTT, 1 mM $CaCl_2$, 1 mM $MgCl_2$, 1 mM 4-(2-aminoethyl)benzenesulfonyl fluoride hydrochloride, protease inhibitor cocktail, and Phosphatase Inhibitor Cocktail 2 and 3. The lysates were rotated for 30 min, subjected to centrifugation at 12,000×g for 15 min, and incubated with anti-FLAG mAb, anti-nectin-4 pAb, or anti-GFP pAb-conjugated Protein A or Protein G Dynabeads at room temperature for 15 min. After the beads were extensively washed with the lysis buffer, bound proteins were eluted by heating at 80°C in an SDS sample buffer (67 mM Tris-HCl at pH 6.8, 2% SDS, 100 mM DTT, 5% sucrose, and 0.005% bromophenol blue) for 2 min, and subjected to SDS-PAGE, followed by Western blotting. The samples separated on SDS-PAGE were transferred to polyvinylidene difluoride membranes (Merck Millipore, Billerica, MA, USA). After being blocked with Block Ace in Tris-buffered saline plus 0.05% Tween 20, the membranes were incubated with the indicated Abs using Can Get Signal Solution 1. After being washed three times with

Tris-buffered saline plus 0.05% Tween 20, the membranes were incubated with horseradish peroxidase-conjugated anti-goat, anti-mouse, anti-rabbit, or anti-rat IgG Ab using Can Get Signal Solution 2. The signals for the proteins were detected using Immobilon Western Chemiluminescent HRP Substrate. The intensity of the bands in Western blotting was calculated using ImageJ 1.48v 32-bit software.

Immunofluorescence microscopy

Confocal images were acquired using a LSM510 META confocal laser-scanning microscope (Carl Zeiss, Jena, Germany) wirth α Plan-FLUAR 100×/1.45 oil objective lens in 2048 × 2048 pixels. The displayed images were applied into maximum signal intensity projection from around 20 confocal images collected at a 0.2 μm step along the z-axis at room temperature.

Protein purification

The reagents for the purification of recombinant proteins are shown in supplementary table 1. FLAG-tagged nectin-4 Ig1/2/3 and the third Ig-like domain of nectin-4, HEK293E cells were transfected with the indicated plasmids. After selection of the transfected cells by puromycin, the cells were cultured in serum-free media. The secreted FLAG-tagged nectin-4 proteins were purified from the conditioned media by DDDDK-Ab-conjugated beads. The DDDDK-peptide-eluted nectin-4 proteins were ultrafiltered several times for dialysis and concentrated using an Amicon Ultra-4 Centrifugal Filter Unit (nominal molecular weight limit 3 kDa, Merck Millipore).

Supplementary Reference

67. Mizushima S, Nagata S. pEF-BOS, a powerful mammalian expression vector. Nucleic Acids Res. 1990; 18:5322.
